# A novel *cis*-element enabled bacterial uptake by plant cells

**DOI:** 10.1038/s41477-025-02161-z

**Published:** 2026-01-02

**Authors:** Chloé Cathebras, Xiaoyun Gong, Rosa Elena Andrade, Ksenia Vondenhoff, Jean Keller, Pierre-Marc Delaux, Makoto Hayashi, Maximilian Griesmann, Martin Parniske

**Affiliations:** 1 Faculty of Biology, Genetics, LMU Munich, Martinsried, Germany; 2 Laboratoire de Recherche en Sciences Végétales, Université de Toulouse, CNRS, UPS, Toulouse, France; 3 Center for Sustainable Resource Science, RIKEN, Yokohama City, Japan

**Keywords:** Rhizobial symbiosis, Phylogenetics

## Abstract

The root nodule symbiosis of plants with nitrogen-fixing bacteria is phylogenetically restricted to a single clade of flowering plants, which calls for as yet unidentified trait acquisitions and genetic changes in the last common ancestor. Here we discovered—within the promoter of the transcription factor gene *Nodule Inception* (*NIN*)—a *cis*-regulatory element (*PACE*), exclusively present in members of this clade. *PACE* was essential for restoring infection threads in *nin* mutants of the legume *Lotus japonicus*. *PACE* sequence variants from root nodule symbiosis-competent species appeared functionally equivalent. Evolutionary loss or mutation of *PACE* is associated with loss of this symbiosis. During the early stages of nodule development, *PACE* dictates gene expression in a spatially restricted domain containing cortical cells carrying infection threads. Consistent with its expression domain, *PACE*-driven *NIN* expression restored the formation of cortical infection threads, also when engineered into the *NIN* promoter of tomato. Our data pinpoint *PACE* as a key evolutionary invention that connected *NIN* to a pre-existing symbiosis signal transduction cascade that governs the intracellular accommodation of arbuscular mycorrhiza fungi and is conserved throughout land plants. This connection enabled bacterial uptake into plant cells via intracellular support structures such as infection threads, a unique and unifying feature of this symbiosis.

## Abstract (web summary)

A key step in the evolution of the nitrogen-fixing root nodule symbiosis, occurring 100 million years ago, subjected the control of *Nodule Inception* (*NIN*) gene expression to a protein complex that regulated transcription much earlier in the arbuscular mycorrhiza symbiosis.

## Main

Nitrogen is essential for plant growth and development^1^. A wide phylogenetic variety of land plants ranging from mosses and gymnosperms to angiosperms have evolved symbioses with nitrogen-fixing bacteria that convert atmospheric nitrogen into ammonium^2^. For example, the fern *Azolla* maintains colonies of nitrogen-fixing cyanobacteria in specialized apoplastic cavities, outside the plant cell wall enclosure^3^. A major biological breakthrough was the evolution of the nitrogen-fixing root nodule symbiosis (RNS) characterized by the intracellular accommodation of bacteria in lateral organs (‘nodules’) formed on roots^4–6^. The occurrence of the RNS is restricted to a monophyletic clade, encompassing four angiosperm orders: the Fabales, Fagales, Rosales and Cucurbitales (FaFaCuRo)^7^. Because of this phylogenetic restriction and scattered occurrence of RNS within the FaFaCuRo, Soltis and colleagues^7^ postulated that the last common ancestor of the FaFaCuRo clade acquired a genetic change, a ‘predisposition’, which enabled members of this clade to subsequently evolve RNS multiple times independently^7^. The intracellular accommodation of bacteria and root nodule development are two genetically separable and, to this extent, independent features of RNS^8,9^. It is therefore genetically possible that they did evolve sequentially and not at the same time. The phylogenetic diversity of bacterial symbionts plus the variation of nodule anatomy and development across the RNS-competent FaFaCuRo species^10,11^ together with the gap of 30 million years between the last common ancestor and the oldest fossil root nodules in this clade^12^ further fuelled the hypothesis that nodule organogenesis evolved several times independently and was not a feature of the last common ancestor^13,14^. The recent discovery of multiple losses of RNS within the FaFaCuRo clade^15,16^ has initiated a discussion about whether this genetic change in the common ancestor was perhaps sufficient for the formation of RNS^17^. Nonetheless, the precise nature of this key event in the evolution of nodulation has remained a mystery for more than two decades^13^.

We asked which evolutionary acquisitions by the last common ancestor, in the form of novel traits and the underlying genetic causes, enabled the evolution of the RNS. From a phylogenetic perspective, such acquisitions should be: (1) exclusively present in the FaFaCuRo clade and absent outside of this clade and (2) conserved throughout the FaFaCuRo clade or at least maintained in RNS-competent (hereafter called ‘nodulating’) species. The uptake of bacteria into living plant cells is, with one exception (*Gunnera*), phylogenetically restricted to the FaFaCuRo clade^4^. The uptake of bacteria requires the localized lysis of the plant cell wall, which threatens cell integrity because of the turgor pressure imposed by the protoplast^6^. A systematic comparison of features associated with the RNS across the entire FaFaCuRo clade pinpoints a single unique and shared trait—the uptake of bacteria into living plant cells with intracellular physical support structures—that fulfils both above-mentioned criteria to be acquired by the common ancestor^6^. These structures come in a diversity of shapes (infection threads (ITs) and infection pegs) and in at least two different cell types (epidermal and cortical) but are all characterized by the apposition of matrix material, which is thought to maintain cell integrity during the localized lysis of the plant cell wall. Although this matrix material is a common feature of all analysed successful bacteria uptake events in FaFaCuRo species, only one type, cortical ITs, can be found in almost all nodulating species^6^. Cortical IT formation is an evolutionary breakthrough because it allowed clonal selection of bacteria^18^, specific control of nutrient exchange and increased nitrogen fixation efficiency^19^. By contrast, in *Gunnera*, cell integrity is maintained by physical closure of a multicellular cavity by extracellular matrix material^20^. This difference, together with the phylogenetic distance of *Gunnera* from the FaFaCuRo clade, suggests an independent origin of bacterial uptake in this genus^6^. To search for gene gains specific for the FaFaCuRo clade, a genome-wide comparative phylogenomic analysis was performed; however, not a single gene following the aforementioned evolutionary pattern was identified^15^.

Here, we tested the hypothesis that the ‘predisposition’ event involved gain of novel *cis*-regulatory elements. Changes in gene regulation can be important drivers of functional and morphological evolution^21,22^. Emergence or loss of even a single *cis*-regulatory element can lead to dramatic phenotypic consequences, for example, novel organ formation^21,22^. Phylogeny has dated the common ancestor of the FaFaCuRo clade to approximately 104 million years ago (Ma)^23,24^. A long-standing hypothesis states that the evolution of RNS involved co-opting genes from the arbuscular mycorrhiza (AM) symbiosis^4,5^, which can be traced back to the earliest land plant fossils 410 Ma (refs. ^25,26^). This hypothesis is underpinned by similarities in intracellular accommodation structures^6^ and the common requirement of both symbioses for a set of so-called common symbiosis genes^5^ that are conserved across land plant species able to form AM and encode symbiotic signal transduction and intracellular restructuring machineries^27–31^.

## Results

### Discovery of *PACE*

**Fig. 1:**
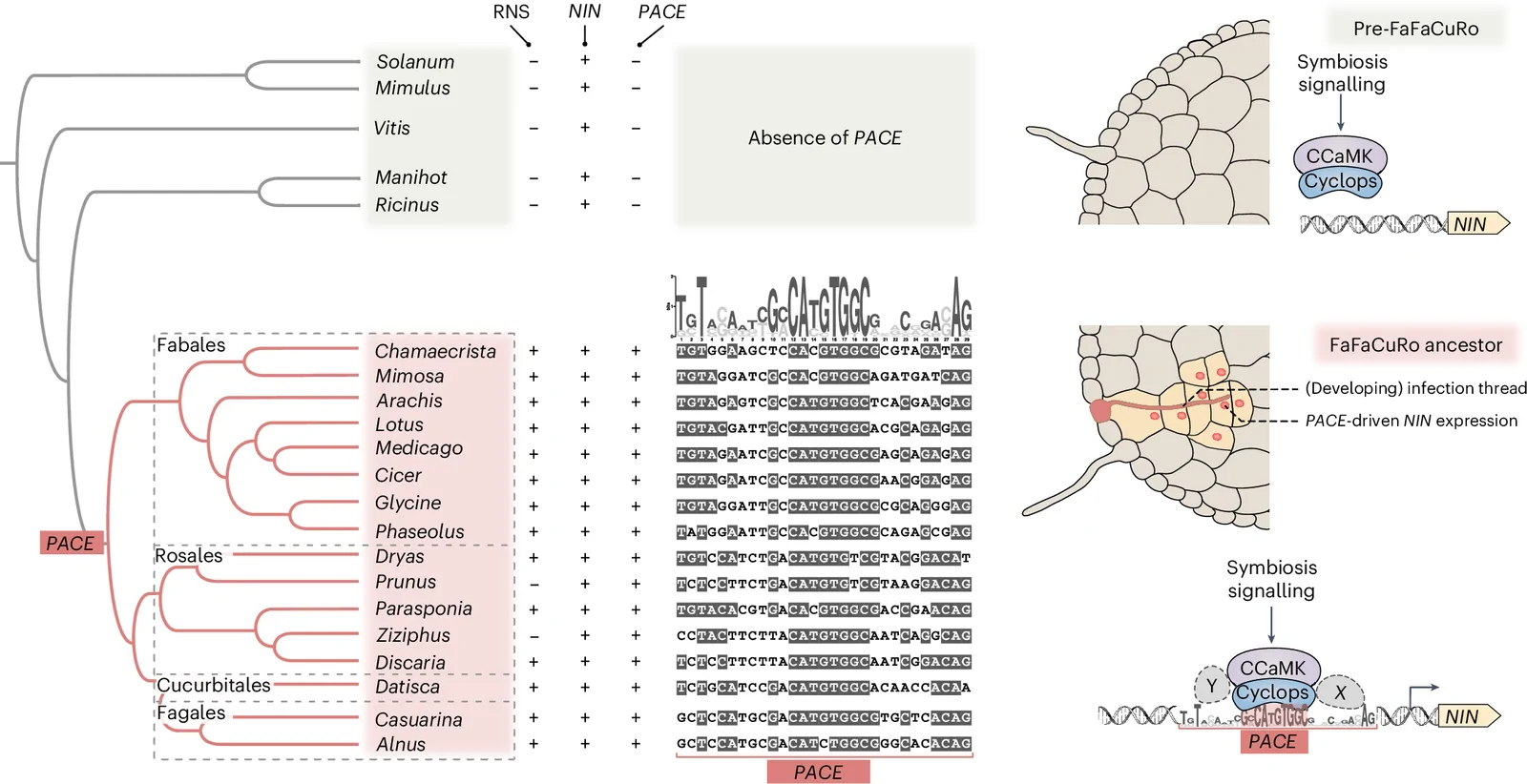
Acquisition of *PACE* was a key step in the evolution of RNS. Left: schematic illustration of the phylogenetic relationships between species inside (light-red shade) and outside (light-grey shade) the FaFaCuRo clade and the presence (+) and absence (−) pattern of RNS, *NIN* and *PACE* (see Extended Data Fig. 1 and Supplementary Fig. 1 for additional data support). Centre: *PACE* sequence alignment of the displayed species, in which grey shadings indicate more than 50% sequence identity. On top of the alignment, the *PACE* consensus sequence is depicted as a position weight matrix calculated from the displayed RNS-competent species. Right: graphical illustration of how *PACE* connected *NIN* to symbiotic transcriptional regulation by CCaMK–Cyclops, enabling IT development in the root cortex. This acquisition coincided with the predisposition event. X and Y represent hypothetical proteins binding to sequences flanking the Cyclops binding site.

**Extended Data Fig. 1:**
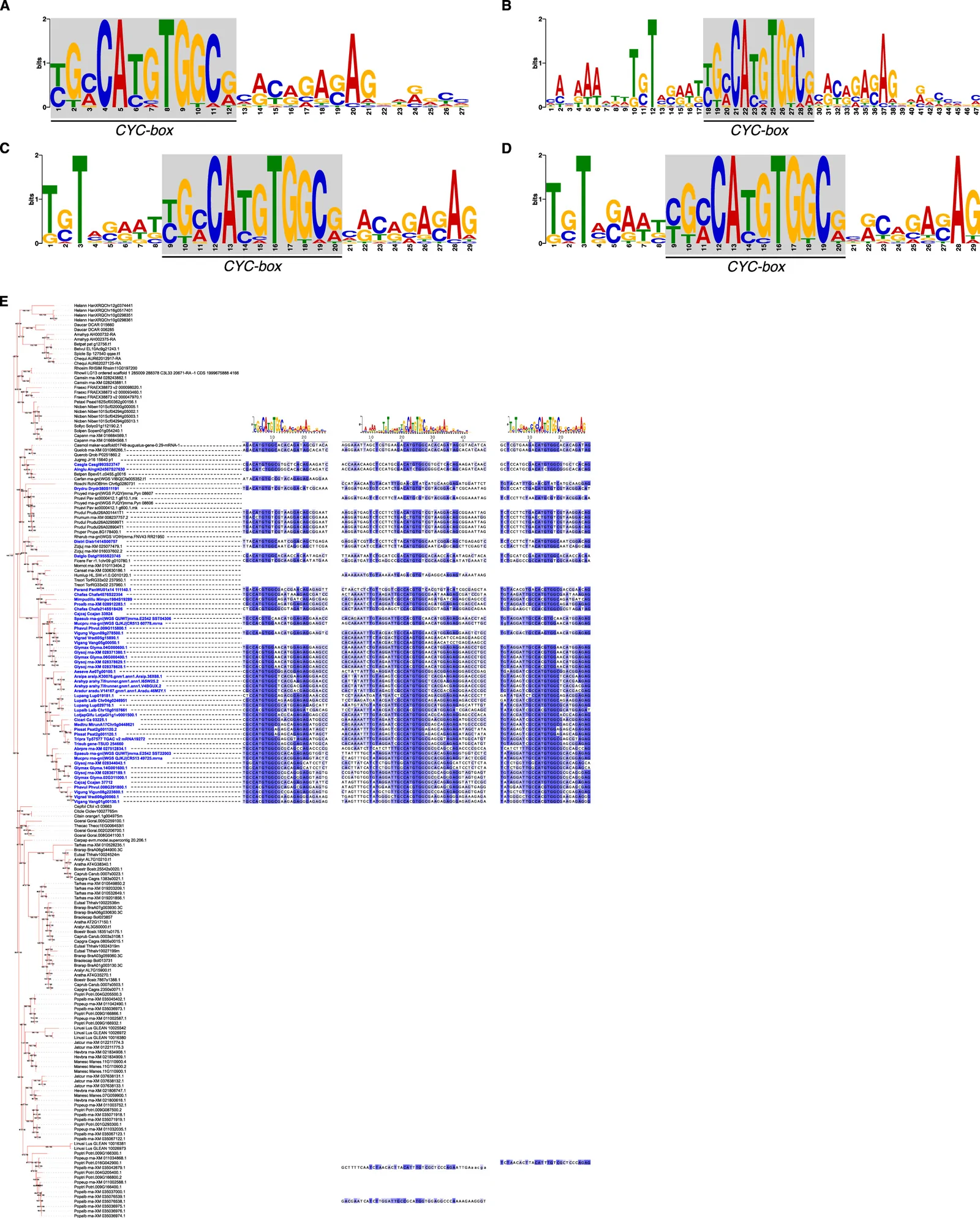
Discovery of *PACE* by MEME analyses. (**A - D**) Consensus sequence of the Position Weight Matrix identified by MEME analyses using the regions upstream of the translational start site (ATG) thus representing the promoters and 5’UTRs of *NIN* and *NLP* genes from 37 angiosperm species (see Methods): (**A**) in a discriminative search for a motif that is present in 3 kb upstream regions of the *NIN* genes from nodulating FaFaCuRo species, but absent in 3 kb upstream regions of the *NIN* genes from species outside of the FaFaCuRo clade and absent in the 3 kb upstream regions of *NLP* genes; (**B**) in an independent, non-discriminative search in the 3 kb upstream regions of the *NIN* genes from nodulating species revealing that the most conserved nucleotides span a larger region than (**A**); (**C**) the resulted most conserved 29 nucleotides derived from upstream regions of *NIN* genes of nodulating FaFaCuRo species; (**D**) the resulted most conserved 29 nucleotides (*PACE*) derived from the upstream region of one representative *NIN* gene per species of nodulating FaFaCuRo species. The Cyclops binding cite (*CYC-box*^34^) is highlighted in grey. (**E**) Motif analyses by FIMO using the upstream regions of the *NIN* and *NLP* genes from an expanded list of 163 species (see Methods). Left: pruned tree from the whole *NLP* tree (demarcated in the black rectangle in Supplementary Fig. 1) corresponding to the *NIN* orthologs. Right: three versions of consensus sequence (left to right, (**A, B** **and D**), respectively) were used to retrieve motifs from the 5 kb upstream region of *NIN* genes *via* FIMO search, the output of which is displayed underneath the consensus sequences. Note that these identified motifs can originate from the + or – strand. The sequences displayed are those with the lowest *q*-value identified by FIMO and this resulted in some cases in overlapping sequences that originated from opposite strands (see Supplementary Table 2). Note that certain nodulating species have multiple copies of the *NIN* gene in their genome, for example *Phaseolus vulgaris* and *Glycine max*. We identified *PACE* in at least one of the *NIN* promoters in each of these species. Sequence names of nodulating species are coloured in blue. Blank lines represent the absence of significant motifs.

The transcription factor-encoding *Nodule Inception* (*NIN*) gene^32,33^ is positioned at the top of an RNS-specific transcriptional regulatory cascade and is indispensable for RNS^32,34,35^. The promoter of *NIN* is a potential physical target for such a co-option event, because it defines the molecular interface between common symbiotic signal transduction and the specific transcriptional networks underlying RNS development^35^. We therefore compared the *NIN*-promoter sequences of 37 angiosperm species including 27 FaFaCuRo members and identified only one motif fulfilling the aforementioned criteria, which we called *Predisposition* *Associated* *cis*-*regulatory* *Element* (*PACE*) (Fig. 1, Extended Data Fig. 1a–d and Supplementary Table 1). The phylogenetic distribution of *PACE* was further investigated in an expanded search comprising 163 plant species in the promoter of *NIN* and the entire *NIN-like protein* (*NLP*) gene family, including *NLP1* from which *NIN* diverged at the base of the eudicots^35^ (Extended Data Fig. 1e, Supplementary Fig. 1 and Supplementary Table 2). *PACE* was found in all nodulating FaFaCuRo members and four non-nodulating species that have lost RNS but maintained *NIN* (Extended Data Fig. 1e and Supplementary Table 3). Importantly, *PACE* was absent from all the *NLP* promoters analysed (Supplementary Fig. 1). Thus, within the *NIN-like* gene family, the phylogenetic distribution of *PACE* is *NIN*- and FaFaCuRo-clade specific and is consistent with a model in which *PACE* was acquired by the *NIN* promoter of the last common FaFaCuRo ancestor. Intriguingly, the 29-nucleotide-long *PACE* encompassed and extended beyond the previously identified binding site of the transcription factor Cyclops, which is encoded by a common symbiosis gene required for the development of both AM and RNS^27,34^ (Fig. 1 and Extended Data Fig. 1).

Given this clade-specific distribution of *PACE*, we searched for conserved motifs in the promoter sequences of two genes encoding transcriptional regulators, *ERF Required for Nodulation 1* (*ERN1*)^36^ (Supplementary Fig. 2) and *Reduced Arbuscular Mycorrhiza 1* (*RAM1*)^37^ (Supplementary Fig. 3) that are also known Cyclops targets. We identified motifs within the promoters of both, *ERN1* and *RAM1*, encompassing the previously identified Cyclops binding sites^36,37^. In sharp contrast to *PACE*, their presence extended beyond the FaFaCuRo clade (Supplementary Figs. 2 and 3).

**Extended Data Fig. 2:**
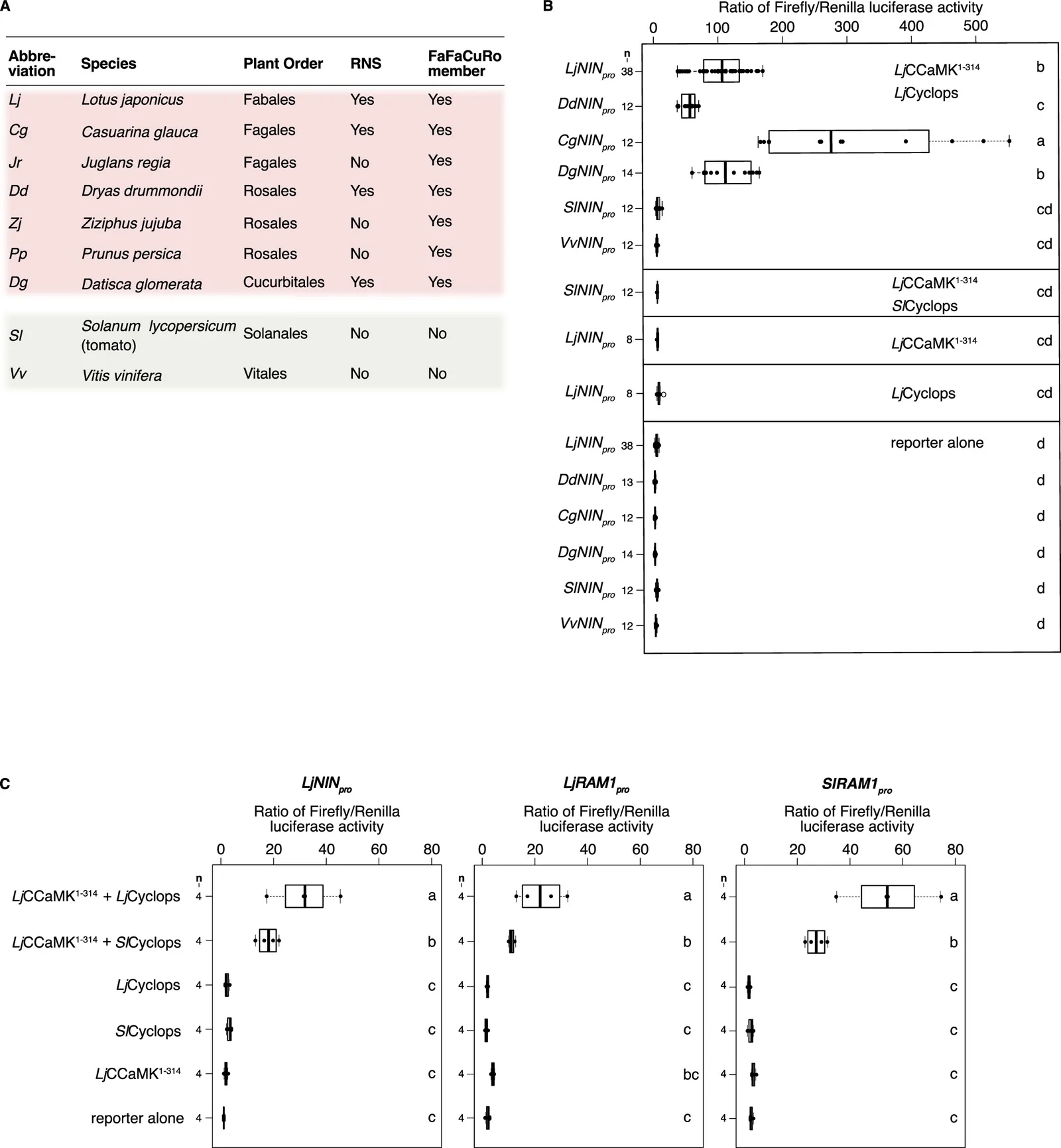
Transcriptional activation of *NIN promoter:Firefly luciferase* reporter gene by CCaMK^1–314^/Cyclops is restricted to *NIN* promoters from species of the FaFaCuRo clade. *Nicotiana benthamiana* leaf cells were transformed with T-DNAs carrying a *Firefly luciferase* reporter gene driven by either of the indicated promoters in tandem with the *AtACT2*_*pro*_:*Renilla luciferase* reporter fusion that provides a quantitative internal standard. (**A**) List of species within the FaFaCuRo clade (light red shade) and outside (light grey shade) and abbreviations. (**B**) Reporter gene activation by *L. japonicus* CCaMK^1–314^/Cyclops via *NIN* promoters (*NIN*_*pro*_) originating from listed species. (**C**) Comparison of the transactivation potential of Cyclops versions from *L. japonicus* and *S. lycopersicum*. Note that the expression of the *Firefly luciferase* reporter gene driven by *LjNIN*_*pro*_, the *RAM1* promoters from *L. japonicus* and *S. lycopersicum* (*LjRAM1*_*pro*_ and *SlRAM1*_*pro*_, respectively) was induced in the presence of CCaMK^1–314^/Cyclops regardless of the origin of Cyclops. In contrast, the transactivation failed with the *SlNIN* promoter (panel (**A**)). Boxplots display the ratio of the Firefly/Renilla luciferase signals. Each dot represents one *N. benthamiana* leaf disc. Thick black lines, median; box, interquartile range; whiskers, lowest and highest data point within 1.5 interquartile range (IQR); black filled circles, data points inside 1.5 IQR; white filled circles, data points outside 1.5 IQR of the upper/lower quartile. The applied statistical method was ANOVA with *post hoc* Tukey: (**B**), *F*_*14,214*_ = 71.07, *p*< 2×10^−16^; (**C**), plots from left to right: *F*_*5,18*_ = 20.58, *p* = 7.14×10^−7^; *F*_*5,18*_ = 25.38, *p* = 1.45×10^−7^ and *F*_*5,18*_ = 40.49, *p* = 3.55×10^−9^, respectively. Different small letters indicate significant differences. Data displayed are from one experiment. Each combination of constructs was tested two times independently with similar outcomes.

**Extended Data Fig. 3:**
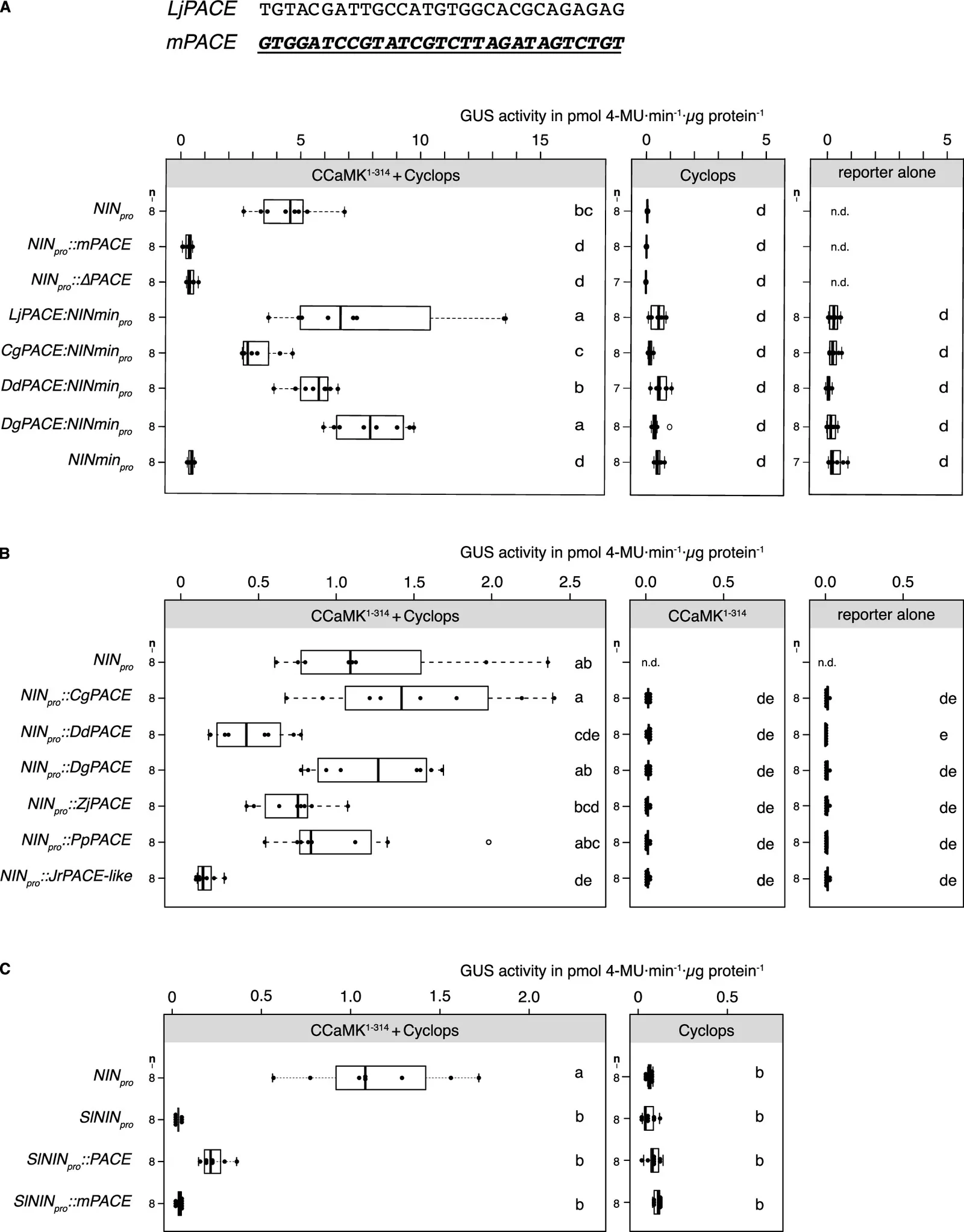
*PACE* sequence variants from species across the FaFaCuRo clade were able to functionally replace *L. japonicus PACE* in a *LjNIN*_*pro*_*:GUS* reporter fusion. *N. benthamiana* leaf cells were transformed with T-DNAs carrying a *GUS* reporter gene driven by either of the indicated promoters: (**A**) the *L. japonicus NIN* promoter (*NIN*_*pro*_), the *LjNIN* promoter with *PACE* mutated or deleted (*NIN*_*pro*_*::mPACE* and *NIN*_*pro*_*::∆PACE*, respectively), or *PACE* sequence variants from the nodulating FaFaCuRo species fused to the *LjNIN* minimal promoter (*NINmin*_*pro*_); (**B**) chimeric promoters where *LjPACE* in the *LjNIN* promoter was replaced with either one of the *PACE* variants from species tested in (**A**) or from non-nodulating FaFaCuRo species including the *Juglans regia PACE-*like motif (*JrPACE-like*); (**C**) the *S. lycopersicum NIN* promoter (*SlNIN*_*pro*_), the *SlNIN* promoter with *LjPACE* (*SlNIN*_*pro*_*::PACE*) or *mPACE* (*SlNIN*_*pro*_*::mPACE*) inserted. For species abbreviations see Extended Data Fig. 2a. Note in (**A**) that the deletion or mutation of *PACE* in *LjNIN* promoter resulted in a drastic reduction in reporter gene expression and in (**C**) insertion of *LjPACE* but not *mPACE* into the *S. lycopersicum* promoter confers transactivation by CCaMK^1−314^/Cyclops. GUS activities are displayed as individual dots in box plots. Each dot represents one *N. benthamiana* leaf disc. Thick black lines, median; box, interquartile range; whiskers, lowest and highest data point within 1.5 interquartile range (IQR); black filled circles, data points inside 1.5 IQR; white filled circles, data points outside 1.5 IQR of the upper/lower quartile. The applied statistical method was ANOVA with *post hoc* Tukey: (**A**) *F*_*20,144*_ = 51.38, *p* < 2×10^−16^; (**B**), *F*_*18,166*_ = 149.1, *p* < 2×10^−16^; (**C**) *F*_*7,62*_ = 30.5, *p* = 7.02×10^−7^. Different small letters indicate significant difference. n.d., not determined. Data displayed are from one experiment. Each combination of constructs was tested two times independently with similar outcomes.

We tested the functional relevance of these distinct phylogenetic distribution patterns in transcriptional activation assays in *Nicotiana benthamiana* leaf cells. Transactivation by Cyclops was restricted to *NIN* promoters from FaFaCuRo species but extended to non-FaFaCuRo species for *RAM1* promoters (Extended Data Fig. 2 and Supplementary Fig. 4). Importantly, *PACE* was necessary and sufficient for the activation of the *NIN* promoter by Cyclops (Extended Data Fig. 3). Together with the exclusive occurrence of *PACE* in the *NIN* promoter of the FaFaCuRo clade, these results are in line with the hypothesis that the mechanistic link between Cyclops and the *NIN* promoter was established in the last common ancestor of this clade (Fig. 1).

### *PACE* drives the expression of *NIN* during IT development in the cortex

*NIN* is indispensable for IT development^32,33^ and its precise spatiotemporal expression is essential for this process^33,38–40^. Because *cis*-regulatory elements are master determinants of gene expression patterns^41^, we investigated the effect of *PACE* on the expression of *NIN* in physical relation to the bacterial uptake and accommodation stages during nodule development. We used the model legume *Lotus japonicus* in combination with its compatible nitrogen-fixing bacterium *Mesorhizobium loti* as experimental system. The process by which *L. japonicus* promotes the intracellular colonization by and accommodation of *M. loti* can be subdivided into successive stages: (1) entrapment of bacteria in a pocket formed by a curled root hair^42^, (2) uptake of bacteria into a developing IT within that root hair^42^, (3) IT progression into and through the outer cortical cell layers^43^, (4) IT branching and extension within the nodule primordium^44^ and (5) release of bacteria from ITs into plant membrane-enclosed organelle-like structures called symbiosomes^44^ leading to (6) mature nodules characterized by infected cells densely packed with symbiosomes and the pink colour of leghemoglobin^45^.

**Extended Data Fig. 4:**
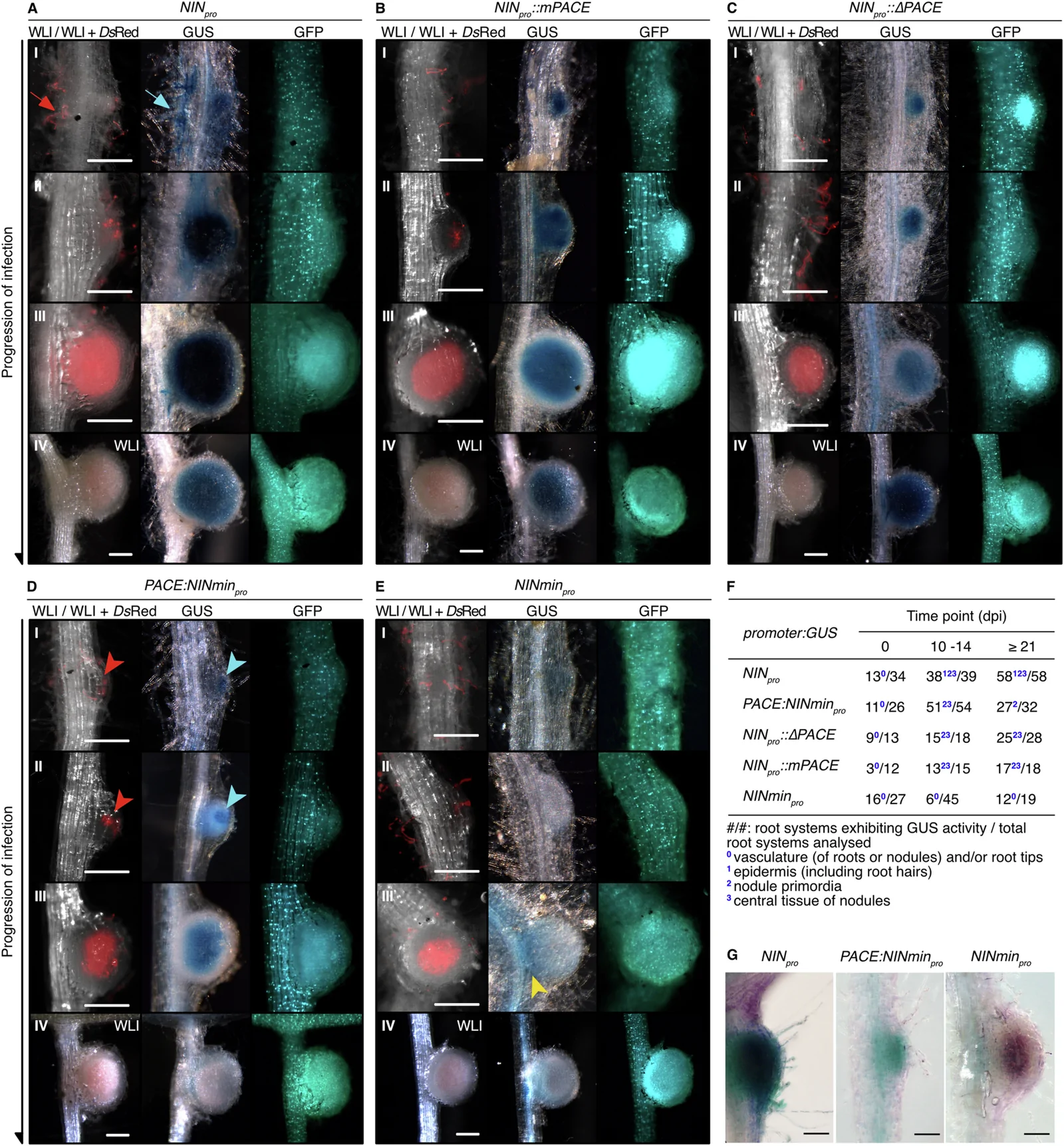
Spatio-temporal *GUS* expression driven by *PACE* and the *NIN* promoter in *L. japonicus* roots during the bacterial infection process. *L. japonicus* wild-type hairy roots were transformed with T-DNAs carrying a *Ubq10*_*pro*_*:NLS-GFP* transformation marker together with a *GUS* reporter gene driven by either of the indicated promoters: (**A**) the 3 kb *LjNIN* promoter (*NIN*_*pro*_); the *LjNIN* promoter with *PACE* (**B**) mutated (*LjNIN*_*pro*_*::mPACE*) or (**C**) deleted (*NIN*_*pro*_*::ΔPACE*); (**D**) *PACE* fused to the *LjNIN* minimal promoter (*PACE:NINmin*_*pro*_) or (**E**) the *LjNIN* minimal promoter (*NINmin*_*pro*_). The progression of bacterial infection was determined by the *Ds*Red signal 10 - 14 days post inoculation (dpi) with *M. loti Ds*Red. Nodules undergoing different stages of infection (panels I to IV) were stained with X-Gluc to reveal the *GUS* expression pattern. Note the overlapping bacterial invasion zone and *PACE:NINmin*_*pro*_*:GUS* expression in early infection stages (red and blue arrowheads in (**D**)) as well as the differences between *PACE:NINmin*_*pro*_*:GUS* and the much broader *NIN*_*pro*_*:GUS* expression at that stage (red and blue arrows in (**A**)). Red arrow and arrowheads: *M. loti Ds*Red. Blue arrow and arrowheads: GUS activity in root hairs bearing ITs and nodule primordia, respectively. The *NINmin*_*pro*_:*GUS* fusion gave only rarely detectable signal, and if so in the vasculature (yellow arrowhead in (**E**)). Only pictures taken under white light illumination (WLI) are displayed for nodules in panel VI to reveal the pink colour of leghemoglobin, characteristic for mature and fully infected nodules. Note that *PACE:NINmin*_*pro*_*:GUS* expression was absent at this stage, whereas the *NIN*_*pro*_*:GUS* resulted in strong blue staining in the nodule regardless of the presence of *PACE* (compare panel IV in (**D**) and (**A - C**)). (**F**) Quantification of transgenic root systems exhibiting *GUS* expression in different cell types and tissues exemplarily displayed in (**A - E**). (**G**) *PACE* drove *GUS* reporter gene expression in the central tissue of primordia and nodules, but was not sufficient for expression in root hairs. Transgenic roots carrying promoter:GUS fusions same as in (**A, D** **and E**) were inoculated with *M. loti lacZ* and dual-stained with X-Gluc and Magenta-Gal. Purple: *M. loti lacZ*. Blue: GUS activity. Note the co-existence of blue and purple staining in root hairs on roots transformed by *NIN*_*pro*_*:GUS*, but not that transformed by *PACE:NINmin*_*pro*_*:GUS*. Data displayed in (**F**) are combined from three independent experiments. Bars, 250 μm.

To determine the *PACE-*mediated spatiotemporal expression domain, we introduced a *GUS* reporter gene driven by *PACE* fused to a region comprising the *NIN* minimal promoter and the 5′ untranslation regions (UTR)^34^ (*PACE:NINmin*_*pro*_*:GUS*) into *L. japonicus* wild-type roots. The roots were subsequently inoculated with *M. loti* MAFF 303099 expressing *Ds*Red (*M. loti Ds*Red) facilitating detection of the bacteria through their fluorescence signal in root hairs and nodules. The *NIN* minimal promoter did not mediate reporter gene expression at any stage of bacterial infection (Extended Data Fig. 4e). Intriguingly, the earliest detectable GUS activity mediated by *PACE:NINmin*_*pro*_*:GUS* was clearly restricted to a zone in the nodule primordia (Extended Data Fig. 4d(I,II)) that roughly correlated with the site of bacterial infection (indicated by a local accumulation of *Ds*Red signal) and later expanded to the entire central tissue of the nodule (Extended Data Fig. 4d(III)). *PACE*-driven reporter expression was neither detected in root hairs harbouring ITs (Extended Data Fig. 4g) nor in nodules in which cells from the central tissue were filled with symbiosomes (Extended Data Fig. 4d(IV)). Importantly, *PACE-*mediated expression was distinct from that mediated by the *LjNIN* 3 kb promoter (*NIN*_*pr*o_) or the *NIN*_*pr*o_ with *PACE* mutated or deleted (*NIN*_*pro*_*::mPACE* and *NIN*_*pro*_*::∆PACE*, respectively) that conferred reporter expression across the central tissue of the nodule (Extended Data Fig. 4a–c(II–IV)). We concluded on the basis of these observations that the *PACE-*mediated expression domain is temporally and spatially restricted and possibly accompanies the development of bacterial accommodation structures in the nodule.

**Fig. 2:**
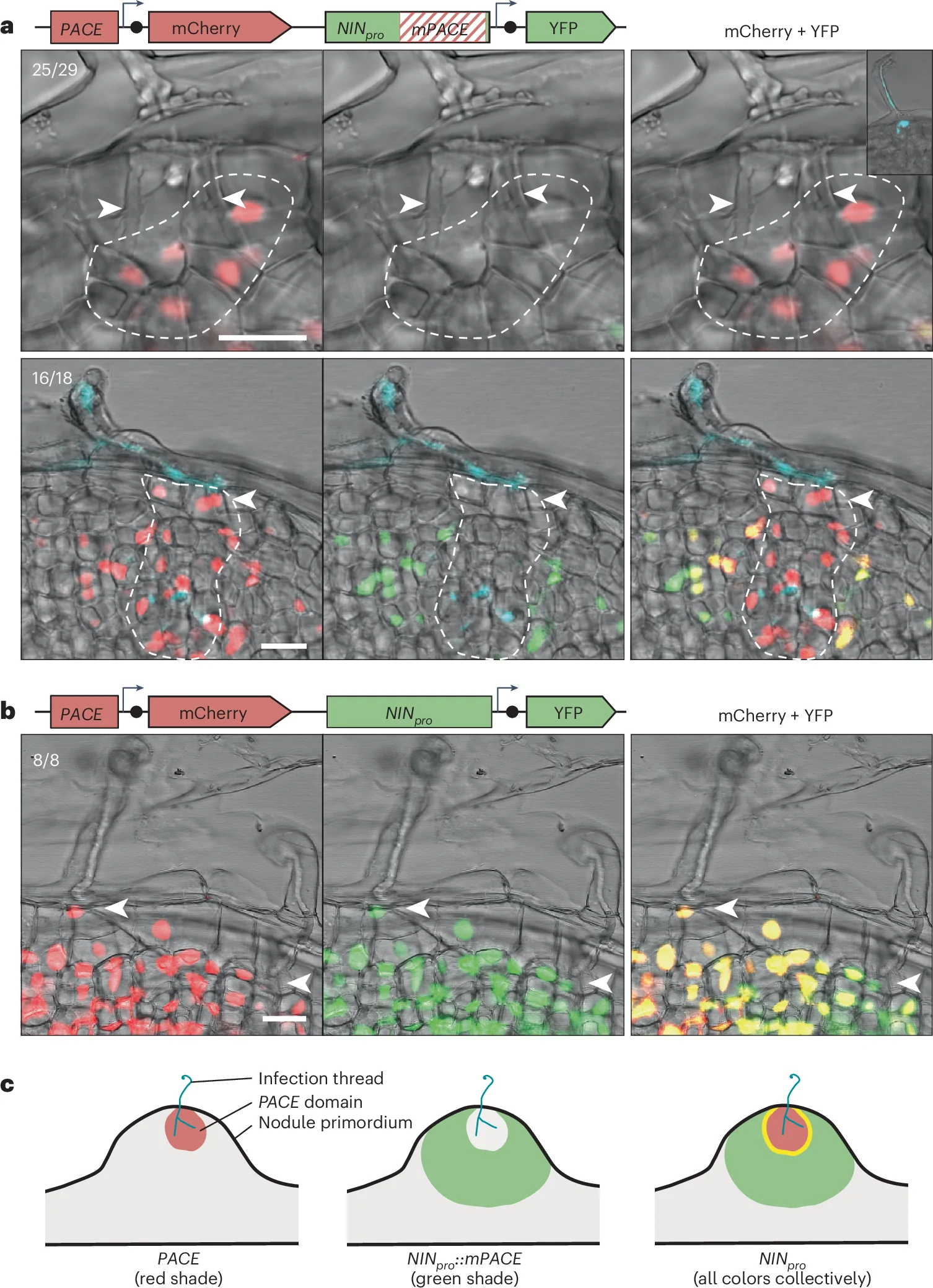
*PACE* drives the expression of *NIN* during IT development in the cortex. **a**,**b**, Sections of representative *L. japonicus* nodule primordia formed upon inoculation with *M. loti* R7A expressing CFP (blue) imaged by confocal laser-scanning microscopy; a comparison of the expression domains determined by *PACE* (*PACE:NINmin*_*pro*_*:NLS-mCherry*; red) and a *NIN* promoter carrying a mutated *PACE* (*NIN*_*pro*_*::mPACE:NLS-YFP*; green) (**a**) or *PACE* (red) and the intact *NIN* promoter (*NIN*_*pro*_*:NLS-YFP*; green) (**b**). The dashed lines demarcate a group of cortical cells in the *PACE* core territory. The arrowheads indicate ITs. Numbers correspond to nodule primordia showing the presented expression pattern/total number of nodule primordia sectioned and inspected. The data are from four independent experiments (see Supplementary Fig. 5 for the first stages of bacterial invasion (stages 2 to 3)). Scale bars, 20 µm. **c**, Graphical interpretation of the expression patterns presented in **a** and **b**. Yellow, overlapping region.

To further resolve this relationship between *PACE*-driven gene expression and bacterial accommodation at the cellular level, we compared—simultaneously in the same tissue—the progression of bacterial infection with the expression pattern mediated by *PACE* fused to the *NIN* minimal promoter (*PACE:NINmin*_*pro*_) and by a *NIN* promoter with mutated *PACE* (*NIN*_*pro*_*::mPACE*). A red and a yellow fluorescent protein (mCherry and YFP, respectively) targeted to the nucleus by fusion to a nuclear localization signal (NLS) were used as reporters. The resulting promoter:reporter fusions (*PACE:NINmin*_*pro*_*:NLS-mCherry* and *NIN*_*pro*_*::mPACE:NLS-YFP*) were placed in tandem on the same transfer-DNA (T-DNA) allowing a nucleus-by-nucleus comparison of their relative expression. This T-DNA construct was introduced into *L. japonicus* wild-type roots that were subsequently inoculated with *M. loti* R7A expressing the cyan fluorescent protein (CFP; Fig. 2) or with *M. loti* MAFF 303099 expressing the green fluorescent protein (GFP; Supplementary Fig. 5) to facilitate detection.

During the first stages of bacterial invasion (stages 2 to 3), *PACE*-mediated *mCherry* was expressed specifically in cortical cells carrying ITs and in directly adjacent cells (Supplementary Fig. 5a). By contrast, the *NIN*_*pro*_*::mPACE*-driven YFP signal was not detected in those cells (Supplementary Fig. 5b). In sections of developing nodules, in which infection had progressed to stage 3 or 4, *PACE*-mediated *mCherry* was expressed specifically in a—hereafter called ‘IT zone’ – comprising cortical cells and primordium cells that carried ITs and in some, but not all, directly adjacent cells^46^ (25 out of 29 nodules inspected; Fig. 2a). Intriguingly, the expression domains marked by mCherry and YFP fluorescence were distinct from each other: whereas the *PACE*-driven mCherry signal was consistently marking the IT zone, the *NIN*_*pro*_*::mPACE*-driven YFP signal was observed in primordium cells surrounding this zone (16 out of 18 nodules inspected; Fig. 2a,c). The thin (approximately 1–2-cell-thick) border between the two domains was characterized by nuclei emitting both YFP and mCherry signals (Fig. 2a). In so-marked cells, ITs were typically not detected. The expression pattern mediated by the *NIN* promoter (containing *PACE*) was congruent with the sum of both promoter fragments (8 out of 8 nodules inspected; Fig. 2b,c).

On the basis of these clearly distinct and complementary reporter expression domains governed by *PACE* versus the remaining promoter, we concluded that (1) *PACE* directs *NIN* expression to a specific IT zone and that (2) the *NIN* promoter comprises *cis*-regulatory elements that drive expression outside the *PACE* territory that is in root hairs (together with *PACE*), non-infected cortical and primordium cells and nodule cells filled with symbiosomes. These additional *cis*-regulatory elements might be addressed by other transcription factors that have been reported to bind to this promoter^47–49^. These transcription factors might be counteracted by, for example, repression in the IT zone.

### Mutational dissection of *PACE* reveals a quantitative effect of sequences flanking the *CYC-box* on IT development

**Fig. 3:**
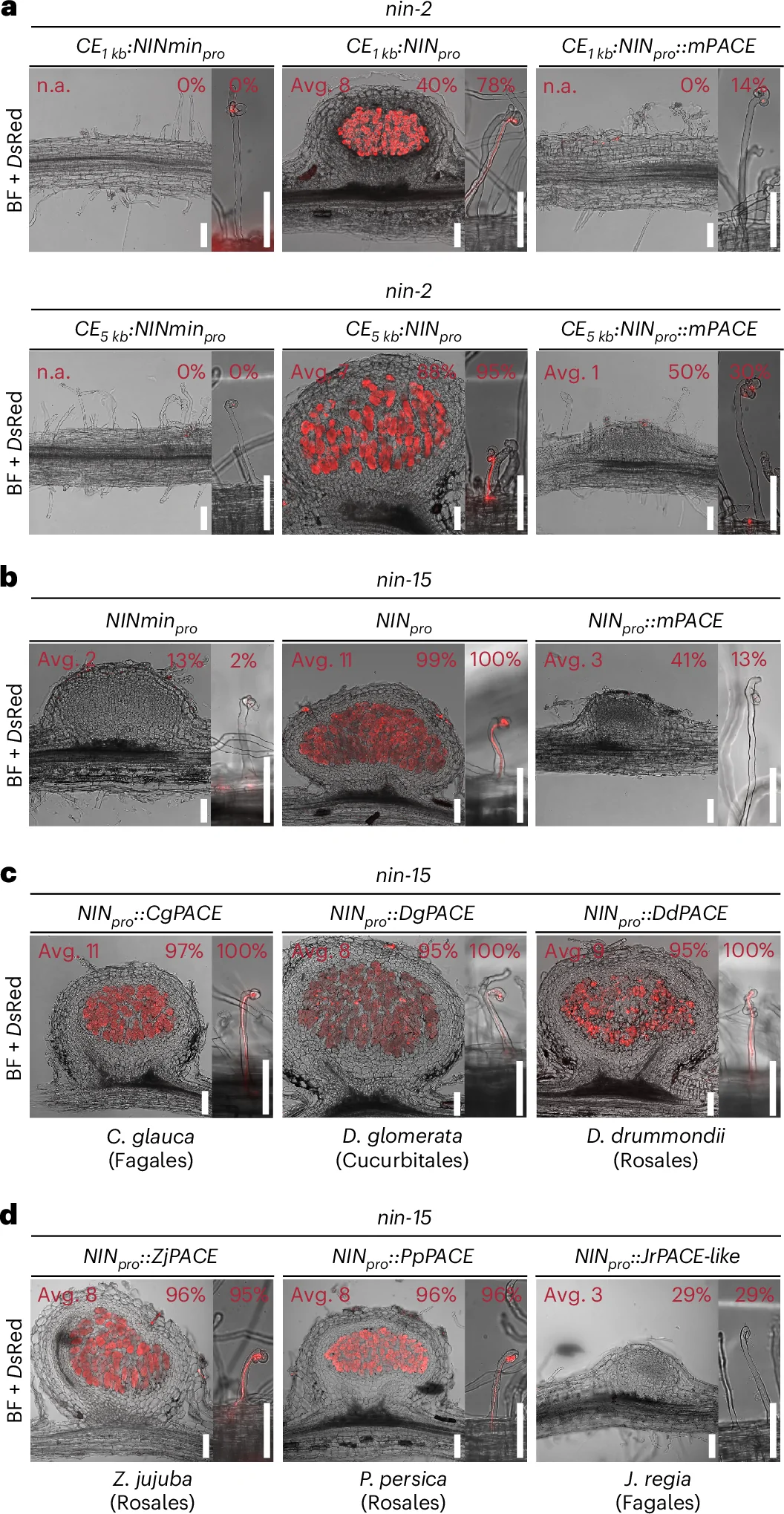
*PACE* is necessary for bacterial infection and functionally conserved across the FaFaCuRo clade. **a**–**d**, Microscopy images of representative nodule sections or root hairs harbouring an IT or an infection pocket from *nin-2* (**a**) or *nin-15* roots (**b**–**d**) transformed with the *LjNIN* gene driven by the indicated promoters (**a**,**b**) and the *L. japonicus NIN* promoter in which *LjPACE* was replaced by *PACE* from nodulating (**c**) and non-nodulating (**d**) FaFaCuRo species, or with a *PACE-like* sequence identified in the *JrNLP1b* promoter. The percentage values of transgenic root systems carrying infected nodules or root hair ITs are indicated. At least five nodules from independent transgenic root systems were sectioned per construct. The percentage of root hair ITs among the total infection events per root pieces and the number of infected nodules per transgenic root system are displayed in Extended Data Figs. 6–9. Scale bars, 100 µm. BF, brightfield; Avg., average number of infected nodules on plants carrying infected nodules; n.a., not applicable.

**Extended Data Fig. 5:**
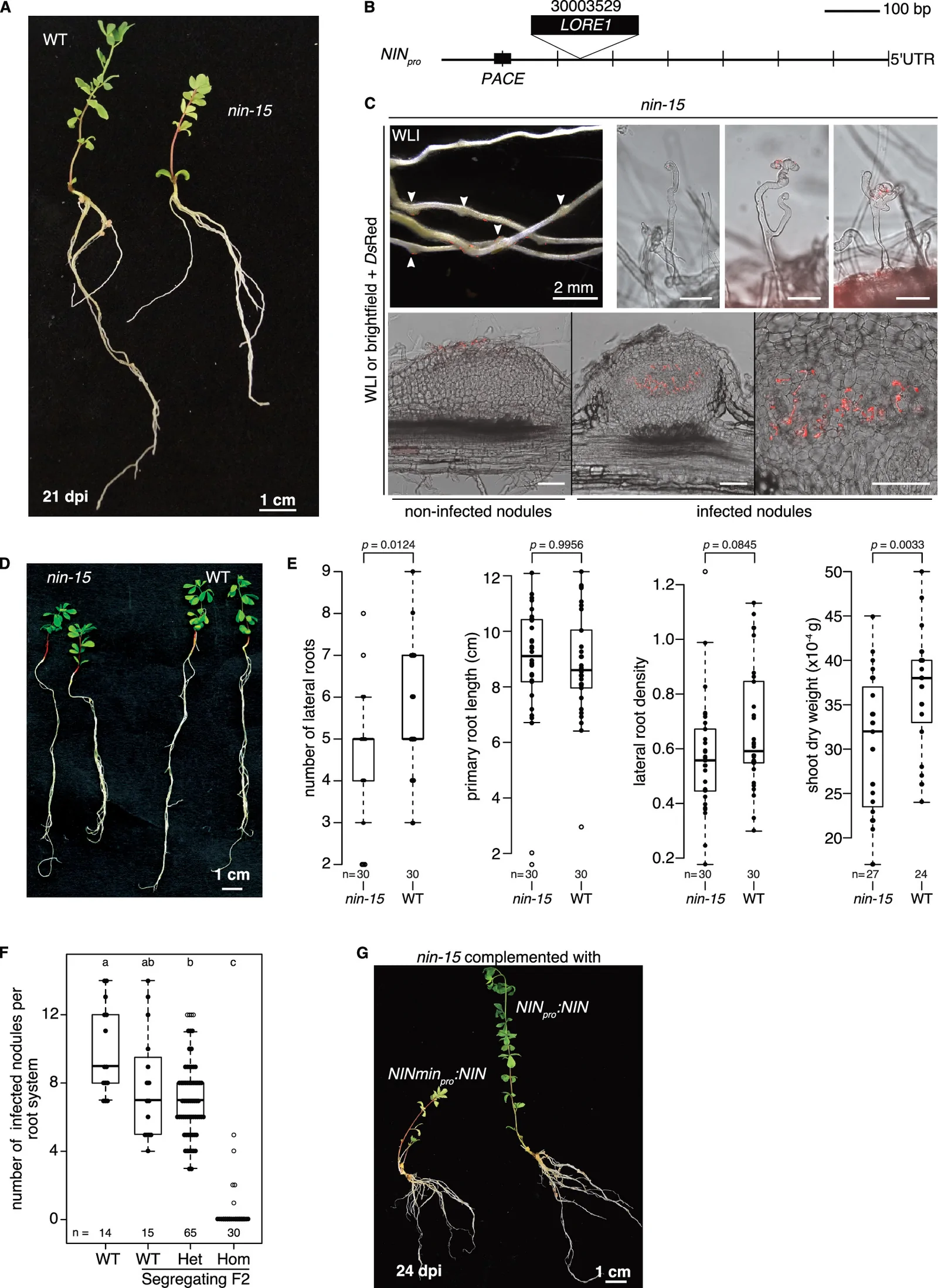
*L. japonicus nin-15* mutant phenotype. (**A**) A representative picture of *L. japonicus* wild-type (WT, left) and *nin-15* (right) plants 21 dpi with *M. loti Ds*Red. (**B**) Position of the *Lotus Retrotransposon 1* (*LORE1*) insertion within the *NIN* promoter in the *nin-15* mutant. (**C**) Representative pictures of *nin-15* root hairs and nodule sections 21 dpi with *M. loti Ds*Red. Forty-nine plants with a total number of 436 nodules were analysed: only four plants bore one or two IT(s) within root hairs and seven plants bore one or two infected nodule(s). Deformed or curled root hairs in the presence of *M. loti Ds*Red were abundant but infection threads were rarely found. Arrowheads: uninfected nodules. Unlabelled bars, 100 μm. (**D – E**) Phenotype of *nin-15* in the presence of a symbiosis-independent nitrogen source (15 mM KNO_3_) for 28 days. (**D**) Pictures documenting the healthy status of *L. japonicus* WT and *nin-15* plants (compare (**D**) and (**A**)) and (**E**) quantitative assessment of parameters displayed in boxplots. Thick black lines, median; box, interquartile range; whiskers, lowest and highest data point within 1.5 interquartile range (IQR); black filled circles, data points inside 1.5 IQR; white filled circles, data points outside 1.5 IQR of the upper/lower quartile. Each dot represents one plant. n: number of plants analysed. Lateral root density: number of lateral roots/primary root length (cm). The applied statistical method was two-tailed Welch’s *t-*test. (**F**) Segregation analysis of *nin-15* assessed by quantifying the number of infected nodules. Each dot in the boxplots represents one plant. n: number of plants analysed. The applied statistical method was ANOVA with *post hoc* Tukey: *F*_*3,120*_ = 84.1, *p* = 2×10^−16^. Different small letters indicate significant difference. (**G**) Representative pictures of *nin-15* plants with hairy roots transformed with the *NIN* gene driven by the *L. japonicus NIN* minimal promoter (*NINmin*_*pro*_) or the 3 kb *NIN* promoter (*NIN*_*pro*_) 24 dpi with *M. loti Ds*Red. Data are from a single experiment. WLI: white light illumination.

To test the relevance and specific role of *PACE* in nodule and IT development, we performed complementation experiments using plants homozygous for the *nin-2* or *nin-15* mutant alleles^32^. The *nin-2* mutant allele harbours a frameshift mutation of the *NIN* gene, leading to a *NIN* loss-of-function phenotype, which is absence of both IT formation and nodule organogenesis^32^, whereas the *nin-15* mutant allele carries a *Lotus Retrotransposon 1* insertion within the *NIN* promoter 143 bp 3′ of *PACE* (Extended Data Fig. 5). We examined the restoration of bacterial infection 21 days post inoculation (dpi) with *M. loti Ds*Red by quantifying the number of root hairs harbouring ITs and the number of infected nodules (Fig. 3 and Supplementary Table 4).

**Extended Data Fig. 6:**
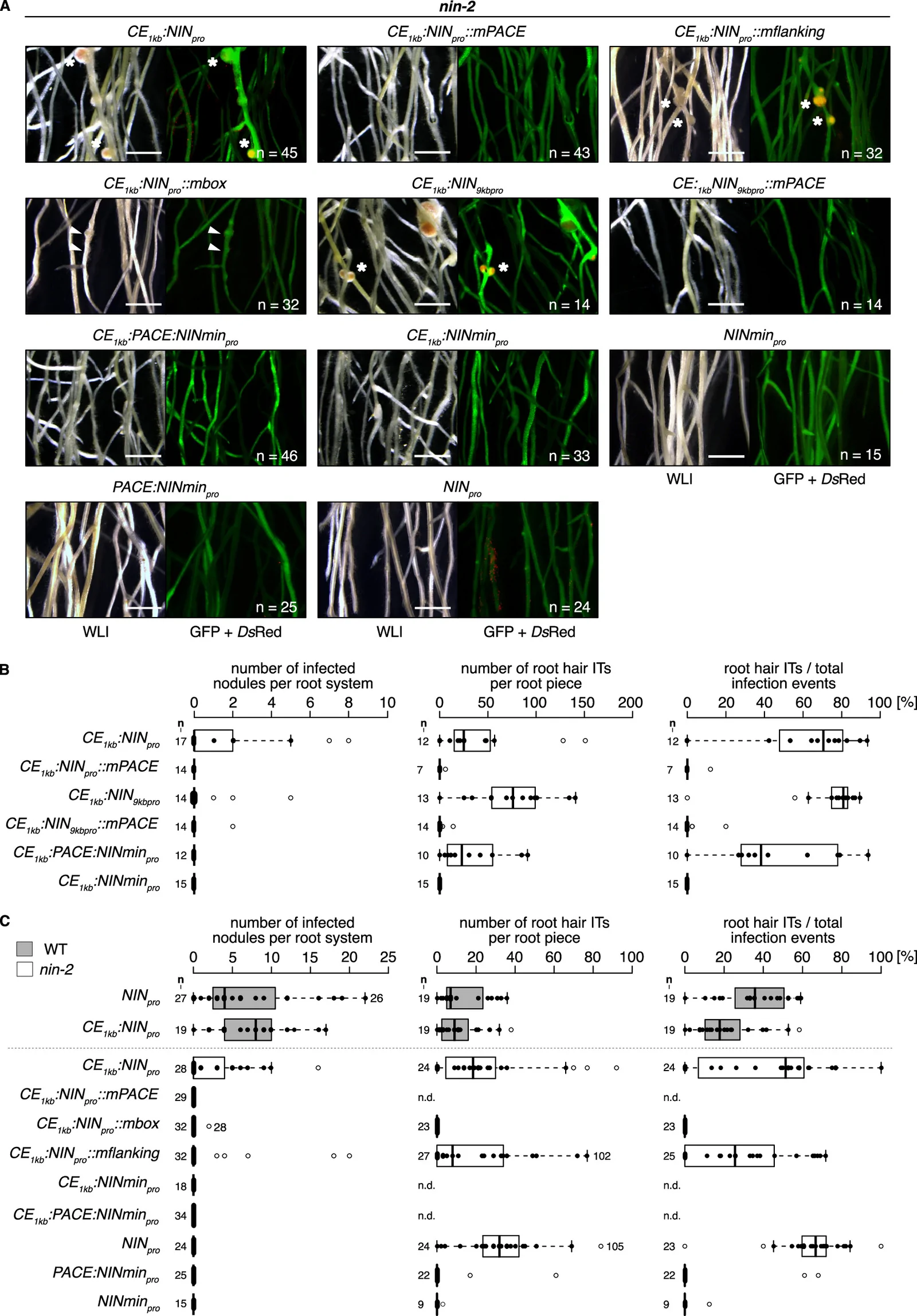
The *CYC-box* and flanking sequences of *PACE* are required for the complete restoration of the bacterial infection process in the *L. japonicus nin-2* mutant. *nin-2* roots were transformed with T-DNAs carrying a *Ubq10*_*pro*_*:NLS-GFP* transformation marker in tandem with the *LjNIN* gene driven by either of the following promoter versions: the cytokinin element-containing region of 1 kb (*CE*_*1kb*_) fused to the 3 kb or 9 kb *LjNIN* promoter (*CE*_*1kb*_*:NIN*_*pro*_ or *CE*_*1kb*_*:NIN*_*9kbpro*_, respectively); *CE*_*1kb*_*:NIN*_*pro*_ or *CE*_*1kb*_*:NIN*_*9kbpro*_ with *PACE* mutated (*CE*_*1kb*_*:NIN*_*pro*_*::mPACE* or *CE*_*1kb*_*:NIN*_*9kbpro*_*::mPACE*, respectively); *CE*_*1kb*_*:NIN*_*pro*_ carrying a mutated Cyclops binding site (*CYC-box*) (*CE*_*1kb*_*:NIN*_*pro*_*::mbox*); *CE*_*1kb*_*:NIN*_*pro*_ carrying mutated sequences flanking the *CYC-box* in *PACE* (*CE*_*1kb*_*:NIN*_*pro*_*::mflanking*); *CE*_*1kb*_ fused to the *LjNIN* minimal promoter (*CE*_*1kb*_*:NINmin*_*pro*_); *CE*_*1kb*_ fused to *PACE* and to *NINmin*_*pro*_ (*CE*_*1kb*_*:PACE:NINmin*_*pro*_); *NIN*_*pro*_, *PACE:NINmin*_*pro*_ or *NINmin*_*pro*_. (**A**) Representative overview pictures of transgenic root systems. Roots were analysed 21 dpi with *M. loti Ds*Red. White asterisks and arrowheads: infected and non-infected nodules, respectively. Bars, 2 mm. (**B – C**) Boxplots displaying the number of root hair ITs or infected nodules and the percentage of root hair ITs among total infection events (sum of bacterial entrapments and ITs). Each dot represents one transgenic *nin-2* root system or root piece. *L. japonicus* WT roots transformed with *NIN*_*pro*_*:NIN* or *CE*_*1kb*_*:NIN*_*pro*_*:NIN* were included as controls. Note the loss of restoration of nodules and IT formation associated with the mutation of *PACE* or only the *CYC-box* in *PACE*; and the reduction of same when sequences flanking the *CYC-box* in *PACE* were mutated. n: number of transgenic root systems or root pieces analysed. Thick black lines, median; box, interquartile range; whiskers, lowest and highest data point within 1.5 interquartile range (IQR); black filled circles, data points inside 1.5 IQR; white filled circles, data points outside 1.5 IQR of the upper/lower quartile. Numbers above the boxplots: the value of individual data points outside of the plotting area. Data are from a single experiment. n.d.: not determined. WLI: white light illumination.

**Extended Data Fig. 7:**
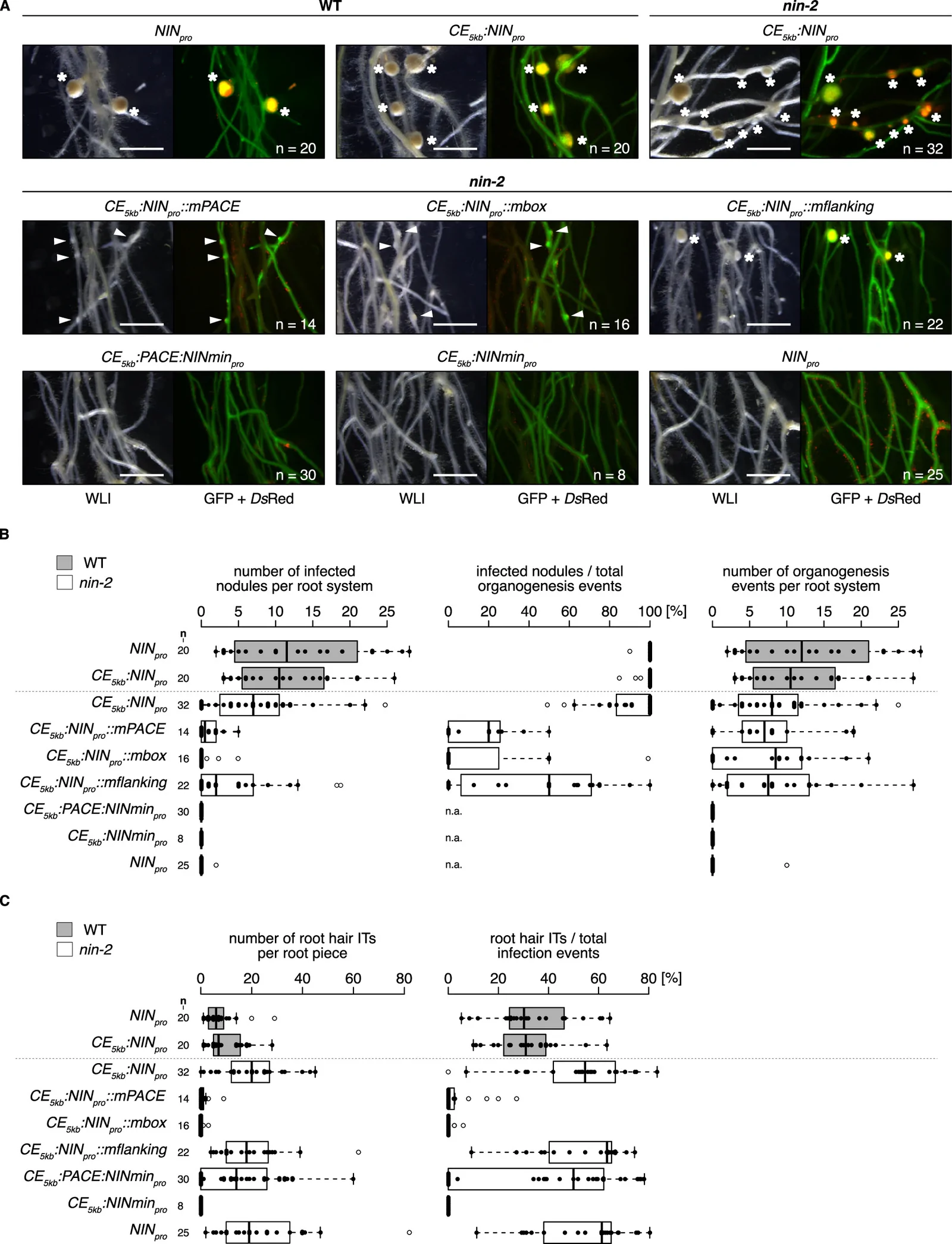
The *CYC-box* and flanking sequences of *PACE* are required for the complete restoration of the bacterial infection process but are dispensable for the nodule organogenesis process in the *L. japonicus nin-2* mutant. *nin-2* roots were transformed with T-DNAs carrying a *Ubq10*_*pro*_*:NLS-GFP* transformation marker in tandem with the *LjNIN* gene driven by either of the following promoter versions: the cytokinin element-containing region of 5 kb (*CE*_*5kb*_) fused to the 3 kb *LjNIN* promoter (*CE*_*5kb*_*:NIN*_*pro*_); *CE*_*5kb*_*:NIN*_*pro*_ with *PACE* mutated (*CE*_*5kb*_*:NIN*_*pro*_*::mPACE*); *CE*_*5kb*_*:NIN*_*pro*_ carrying a mutated Cyclops binding site (*CYC-box*) (*CE*_*5kb*_*:NIN*_*pro*_*::mbox*); *CE*_*5kb*_*:NIN*_*pro*_ carrying mutated sequences flanking the *CYC-box* in *PACE* (*CE*_*5kb*_*:NIN*_*pro*_*::mflanking*); *CE*_*5kb*_ fused to the *LjNIN* minimal promoter (*CE*_*5kb*_*:NINmin*_*pro*_); *CE*_*5kb*_ fused to *PACE* and to *NINmin*_*pro*_ (*CE*_*5kb*_*:PACE:NINmin*_*pro*_) or *NIN*_*pro*_. (**A**) Representative overview pictures of transgenic root systems. Roots were analysed 21 dpi with *M. loti Ds*Red. White asterisks and arrowheads: infected and non-infected nodules, respectively. Bars, 2 mm. (**B**) Boxplots displaying the number of infected nodules, the percentage of infected nodules among total organogenesis events (sum of infected and non-infected nodules) and the number of organogenesis events. (**C**) Boxplots displaying the number of root hair ITs and the percentage of root hair ITs among total infection events (sum of bacterial entrapments and ITs). Each dot represents one transgenic *nin-2* root system or root piece. *L. japonicus* WT roots transformed with *NIN*_*pro*_*:NIN* or *CE*_*5kb*_*:NIN*_*pro*_*:NIN* were included as controls. Note that the mutation of *PACE* or only the *CYC-box* in *PACE* led to an almost complete loss of IT formation and infected nodules per root system while nodule organogenesis was not significantly reduced; and that mutation of sequences flanking the *CYC-box* in *PACE* led to a reduction of the number of infected nodules per root systems. n: number of transgenic root systems or root pieces analysed. Thick black lines, median; box, interquartile range; whiskers, lowest and highest data point within 1.5 interquartile range (IQR); black filled circles, data points inside 1.5 IQR; white filled circles, data points outside 1.5 IQR of the upper/lower quartile. Data are from a single experiment. n.a.: not applicable. WLI: white light illumination.

**Extended Data Fig. 8:**
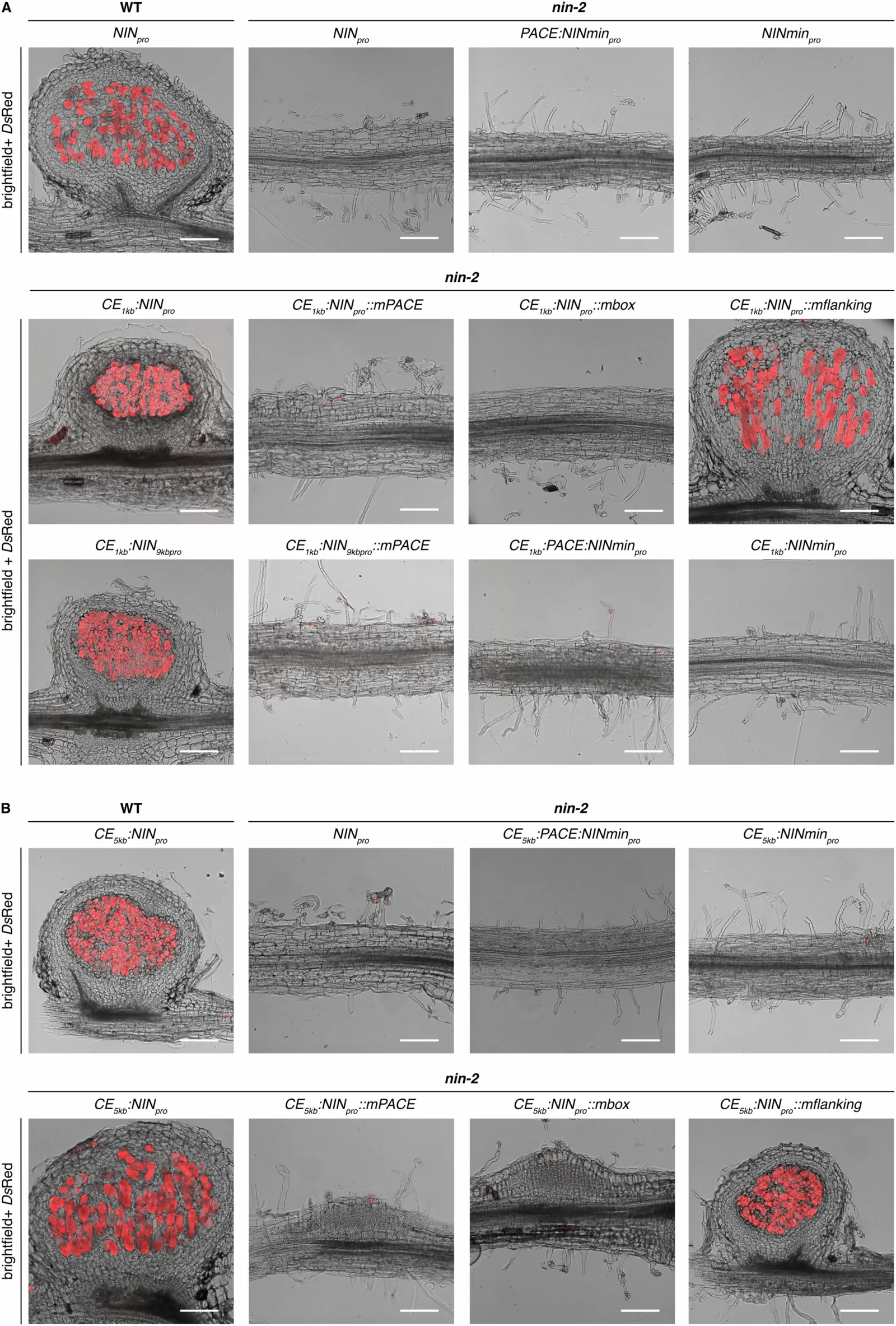
The *CYC-box* and flanking sequences of *PACE* are required for the complete restoration of the bacterial infection process but are dispensable for the nodule organogenesis process in the *L. japonicus nin-2* mutant. Pictures of nodule sections or roots from *L. japonicus nin-2* roots 21 dpi with *M. loti Ds*Red from the same experiments depicted in Extended Data Fig. 6 (**A**) and Extended Data Fig. 7 (**B**). Nodule sections from *L. japonicus* WT roots transformed with *NIN*_*pro*_*:NIN* and *CE*_*5kb*_*NIN*_*pro*_*:NIN* were included for comparison. Note that when the cytokinin element-containing region of 1 kb was fused to *NIN*_*pro*_ nodule organogenesis was abolished by mutation of *PACE* or only the *CYC-box* in *PACE* and that these mutations did not abolish organogenesis when the cytokinin element-containing region of 5 kb was fused to *NIN*_*pro*_. At least five nodules from independent transgenic root systems were sectioned per construct. Bars, 100 μm.

Nodule development in the legume *Medicago truncatula* is dependent on *NIN* expression mediated by a regulatory region containing several putative cytokinin responsive elements (*CE*)^40^. In *L. japonicus*, a similar *CE* region is positioned 45 kb upstream of the *NIN* transcriptional start site^40^. To enable transgenic complementation experiments, we synthetically fused a 1 kb or 5 kb region encompassing this distant *CE* to the 5′ end of a 3-kb *NIN* promoter. The *NIN* gene driven by these promoters (*CE*_*1kb*_*:NIN*_*pro*_*:NIN* and *CE*_*5kb*_*:NIN*_*pro*_*:NIN*) restored the formation of root hair ITs on 78% and 95% and infected nodules on 40% and 88% of *nin-2* transgenic root systems, respectively (Fig. 3a, Extended Data Figs. 6–8 and Supplementary Figs. 6 and 7). Importantly, this complementation success relied on the presence of *PACE*. *nin-2* roots transformed with the same fusion design but carrying a mutation of *PACE* (*CE*_*1kb*_*:NIN*_*pro*_*::mPACE:NIN* and *CE*_*5kb*_*:NIN*_*pro*_*::mPACE:NIN*) did not restore root hair ITs; however, nodule formation was not impaired when using the cytokinin element-containing region of 5 kb (*CE*_*5kb*_*:NIN*_*pro*_*::mPACE:NIN*). We concluded that *PACE* is indispensable for bacterial infection but not for nodule development.

The 29-bp-long *PACE* sequence revealed by MEME encompasses and extends beyond the previously identified Cyclops binding site (*CYC-box*^34^, ‘box’; Extended Data Fig. 1). Its degree of conservation may be interpreted as a trace of an ancestral *PACE* version present in the last common ancestor of the FaFaCuRo clade. Within *PACE*, the *CYC-box* is surrounded by less conserved flanking sequences. To dissect the specific contributions of the *CYC-box* and *PACE* sequences flanking the *CYC-box* (‘flanking’) to *PACE* function, we mutated the box and the flanking sequences independently (*CE:NIN*_*pro*_*::mbox:NIN* and *CE:NIN*_*pro*_*::mflanking:NIN*, respectively). Mutation of the *CYC-box* abolished root hair ITs. Interestingly, mutation of the flanking sequences led to a 50% reduction of the number of transgenic root systems carrying infected nodules, whereas the formation of root hair ITs was not impaired (Extended Data Figs. 6–8 and Supplementary Figs. 6and 7). This mutational dissection revealed two separable functions of *PACE*: whereas the *PACE*–Cyclops connection is essential for IT development, the flanking sequences significantly promote bacterial infection during nodule development and possibly act as binding sites for additional, yet undefined, transcription factors (conceptually labelled X and Y in Fig. 1). Our data suggest that *PACE* comprises synergistic binding sites for both Cyclops and cooperating transcription factors. We conclude that the high level of conservation of the *CYC-box* is a consequence of the indispensable nature of this *cis*-element for the progression of the IT through the cortex. The higher level of diversification of sequences flanking the *CYC-box* might be a consequence of changes in transcription factors occupancy over evolutionary time scales. Considering this scenario, it is possible that such flanking sequence-occupying transcription factors are not conserved throughout the entire FaFaCuRo clade.

**Fig. 4:**
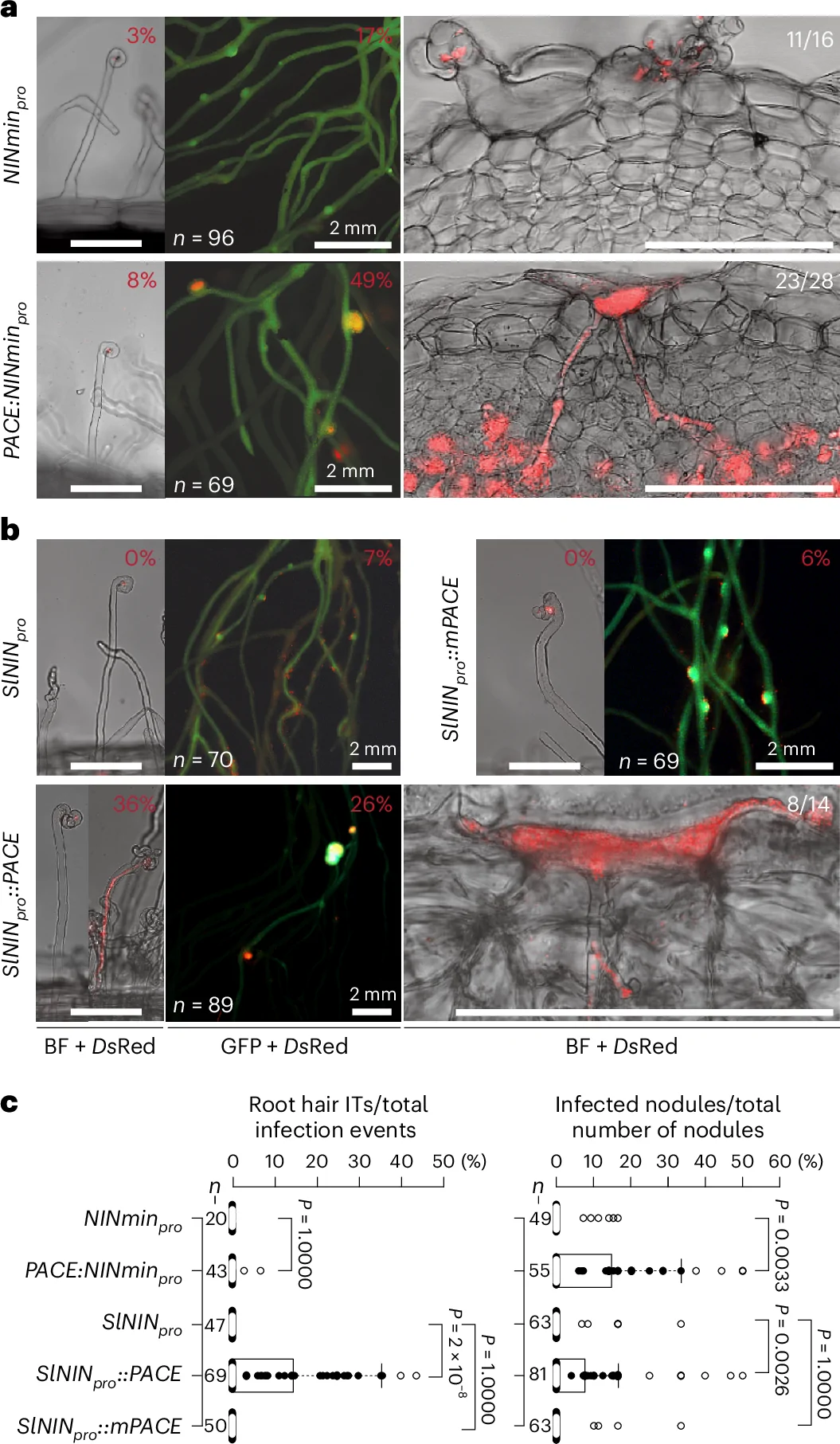
*PACE* enables IT formation in the cortex. **a**,**b**, Representative pictures of *nin-15* root hairs, root and nodule sections (see Extended Data Fig. 10 for overview pictures) transformed with the *L. japonicus NIN* gene driven by *NINmin*_*pro*_ or *PACE:NINmin*_*pro*_(**a**) or *SlNIN*_*pro*_ and *SlNIN*_*pro*_ with *LjPACE* or *mPACE* inserted (**b**). The percentage values of transgenic root systems carrying root hair ITs or infected nodules are indicated. Ratios indicate the number of nodules showing the presented pattern/total number of nodules sectioned and inspected. The data in **a** are from two independent experiments (see Extended Data Fig. 10 for second replicate), and the data in **b** are from a single experiment. **c**, Box plots displaying the percentage of root hair ITs and infected nodules per transgenic root system. The thick white lines represent the median; the box represents the IQR; whiskers represent the lowest and highest data point within 1.5× IQR; black-filled circles represent the data points inside 1.5× IQR; white-filled circles represent the data points outside 1.5× IQR of the upper quartile. The statistical method applied was the two-tailed Fisher’s exact test. The data in **c** are from **a** and **b**. Unlabelled scale bars, 100 µm. *n*, number of transgenic root systems or root pieces analysed. BF, brightfield.

**Extended Data Fig. 9:**
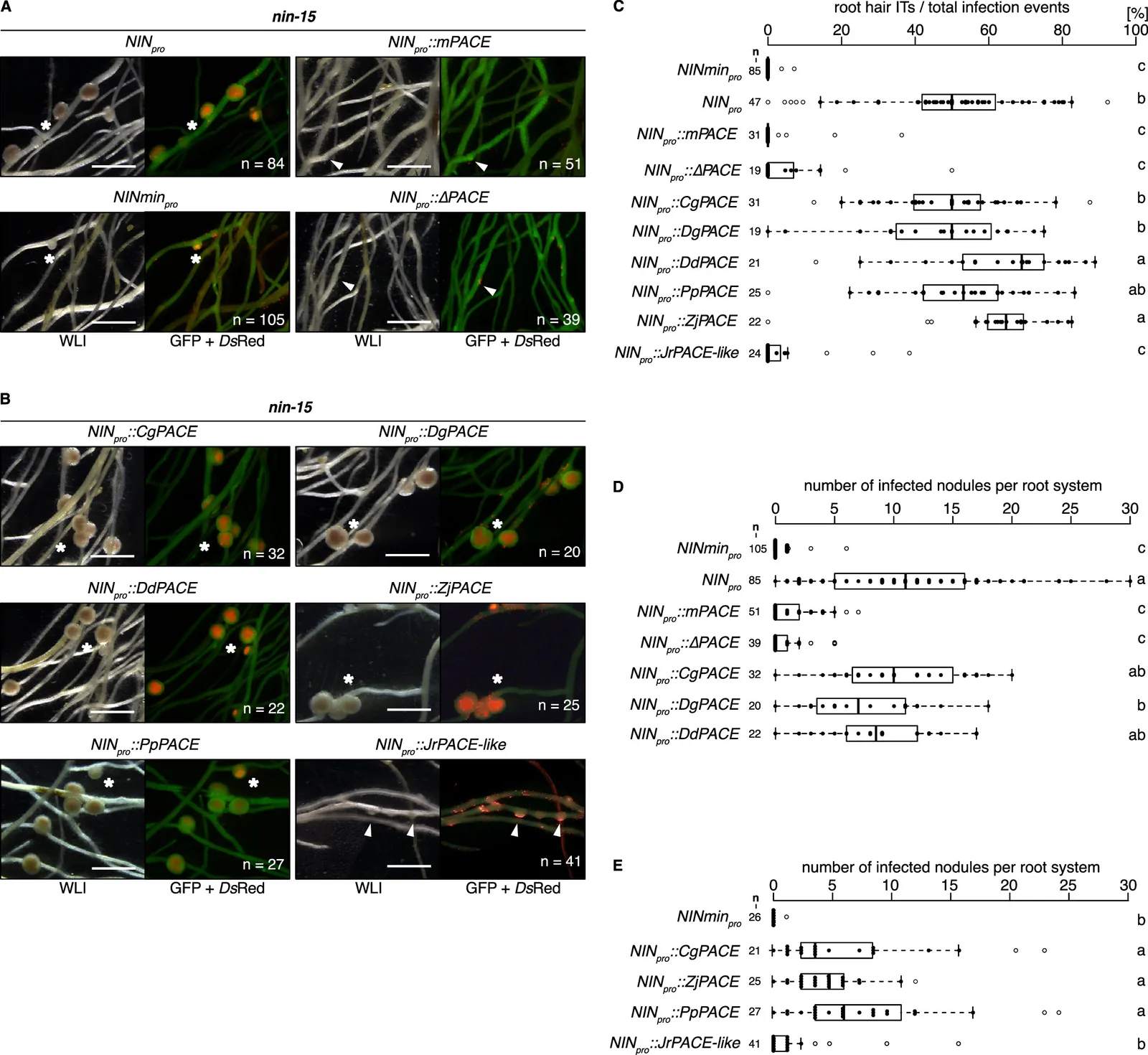
*PACEs* from FaFaCuRo species are functionally equivalent in restoring bacterial infection in the *L. japonicus nin-15* mutant. *L. japonicus nin-15* roots were transformed with T-DNAs carrying a *Ubq10*_*pro*_*:NLS-GFP* transformation marker in tandem with the *LjNIN* gene driven by either of the following promoters: (**A**) the 3 kb *LjNIN* promoter (*NIN*_*pro*_), the *LjNIN* minimal promoter (*NINmin*_*pro*_), the 3 kb *LjNIN* promoter with *PACE* deleted (*NIN*_*pro*_*::∆PACE*) or mutated (*NIN*_*pro*_*::mPACE*); (**B**) the 3 kb *LjNIN* promoter with *LjPACE* replaced with either of the *PACE* sequence variants from nodulating or non-nodulating FaFaCuRo species and analysed 21 dpi with *M. loti Ds*Red. (**A – B**) Representative overview pictures of *nin-15* transgenic roots systems. Sections of representative nodules are displayed in Fig. 3. Note the drastic reduction of restoration of infection in nodules and root hairs associated with the mutation or deletion of *PACE* as well as the replacement of *PACE* with *JrPACE-like* in the context of the *LjNIN* promoter. White asterisks and arrowheads: infected and non-infected nodules, respectively. (**C – E**) Boxplots displaying (**C**) the percentage of root hair ITs among total infection events (sum of bacterial entrapments and ITs) and (**D – E**) the number of infected nodules from two independent experiments. Each dot represents one *nin-15* transgenic root piece (**C**) or root system (**D – E**). (**C**) displays merged data from experiments in (**D – E**) as the percentage represents a normalised value calculated for each root piece (see Supplementary Table 4). Thick black lines, median; box, interquartile range; whiskers, lowest and highest data point within 1.5 interquartile range (IQR); black filled circles, data points inside 1.5 IQR; white filled circles, data points outside 1.5 IQR of the upper/lower quartile. n: number of transgenic root systems or root pieces analysed. For species abbreviations see Extended Data Fig. 2a. The applied statistical method was ANOVA with *post hoc* Tukey: (**C**) *F*_*9,313*_ = 106.7, *p* < 2×10^−16^; (**D**) *F*_*6,346*_ = 82.89, *p* < 2×10^−16^; (**E**) *F*_*4,135*_ = 20.18, *p* = 4.76×10^−13^. Different small letters indicate significant differences. Data are from a single experiment. Bars, 2 mm. WLI: white light illumination.

**Extended Data Fig. 10:**
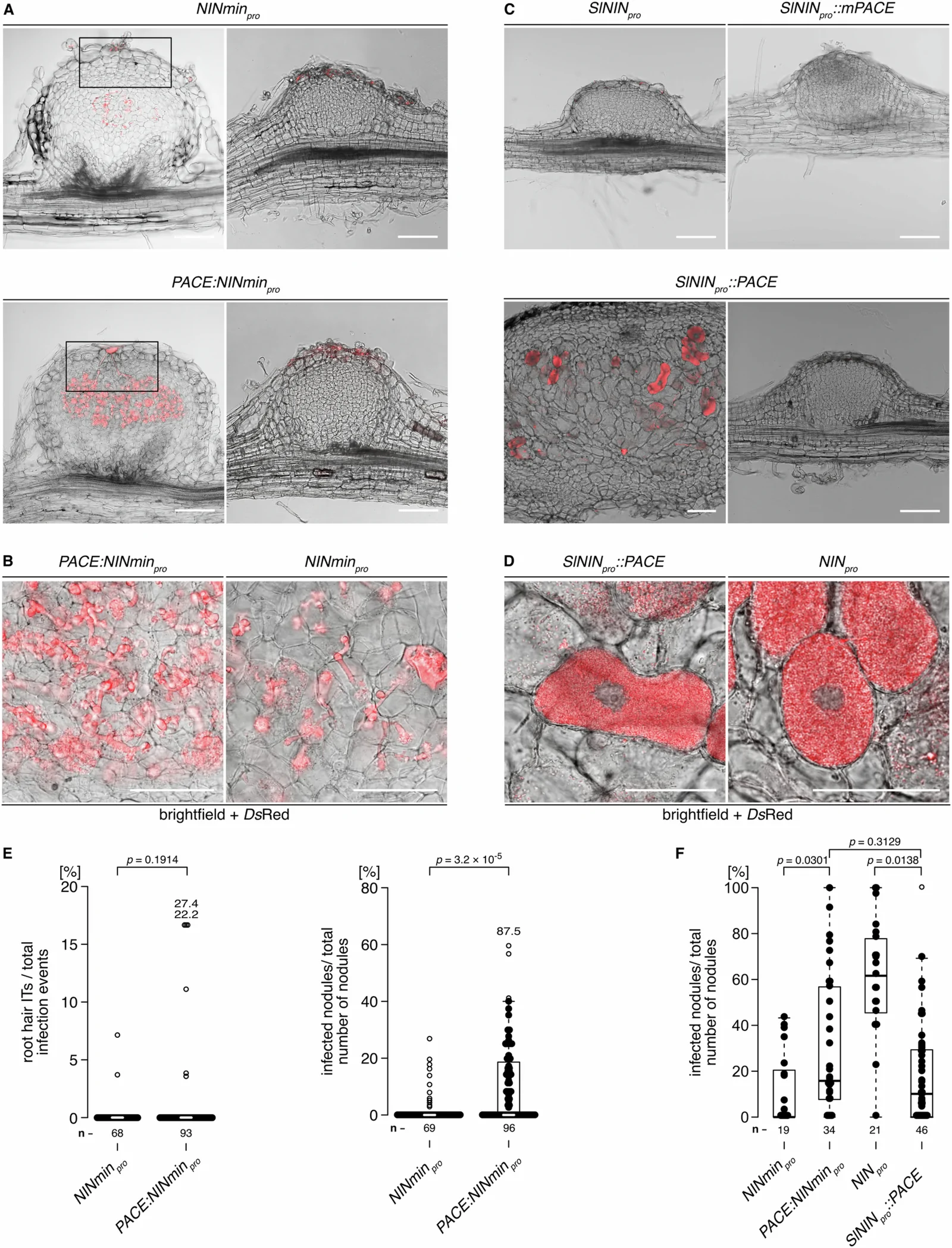
*PACE* alone or in the context of the *S. lycopersicum NIN* promoter (a species outside of the FaFaCuRo clade) enables IT formation in the cortex. (**A – D**) Representative pictures of sections of nodules formed on *L. japonicus nin-15* roots transformed with T-DNAs carrying a *Ubq10*_*pro*_*:NLS-GFP* transformation marker together with the *LjNIN* gene driven by either of the following promoters: (**A – B**) the *L. japonicus NIN* minimal promoter (*NINmin*_*pro*_) or *PACE* fused to *NINmin*_*pro*_ (*PACE:NINmin*_*pro*_); (**C – D**) the 3 kb *S. lycopersicum NIN* promoter (*SlNIN*_*pro*_), the 3 kb *SlNIN* promoter with mutated *PACE* (*SlNIN*_*pro*_*::mPACE*) or with *L. japonicus PACE* inserted (*SlNIN*_*pro*_*::PACE*), 21 dpi with *M. loti Ds*Red (from the same experiments depicted in Fig. 4). Black rectangles in (**A**) demarcate the enlarged area displayed in Figs. 4a and 4b to focus on the initial infection structures. Note the absence of cells filled with symbiosomes in nodules transformed with the *LjNIN* gene driven by *PACE:NINmin*_*pro*_ or *NINmin*_*pro*_. By contrast, infected cells were often filled with symbiosomes in the *SlNIN*_*pro*_*::PACE:NIN-*transformed nodules, like those resulted by *NIN*_*pro*_*:NIN* (see (**C**) and compare the two sections in (**D**)). (**E – F**) Boxplots displaying the percentage of root hair ITs among total infection events (sum of bacterial entrapments and ITs) or the percentage of infected nodules among total number of nodules (**E**) 21 dpi and (**F**) 35 dpi with *M. loti Ds*Red, respectively. Each dot represents one *nin-15* transgenic root system or root piece. (**E**) displays results from an independent repetition from the experiment depicted in Fig. 4. n: number of transgenic root systems or root pieces analysed. Thick white (**E**) and black (**F**) lines, median; box, interquartile range; whiskers, lowest and highest data point within 1.5 interquartile range (IQR); black filled circles, data points inside 1.5 IQR; white filled circles, data points outside 1.5 IQR of the upper/lower quartile. Data displayed are from a single experiment. Numbers above the boxplots: the value of individual data points outside of the plotting area. The applied statistical method was two-tailed Fisher’s exact test. Bars, (**A** and **C**) 100 μm; (**B** and **D**) 50 μm.

*PACE*-mediated *NIN* expression defined an infection zone in the nodule cortex (Fig. 2). To genetically separate the initiation of nodule development from IT formation and thereby enable a focused analysis of the role of *PACE* in cortical IT formation, we utilized the *nin-15* mutant, which is impaired in IT formation but retains the capacity to form nodules. Most of these nodules were uninfected (92% and 86% plants carrying no root hair ITs and no infected nodules, respectively), and cortical cells filled with symbiosomes were never observed (Extended Data Fig. 5). This mutant therefore provided an ideal background to study the role of *PACE* in cortical IT formation, circumventing the negative epistatic effect of the inability of *nin* loss-of-function mutants to initiate cell divisions^32,38–40,50^ (Figs. 3b–d and 4, Extended Data Figs. 9 and 10 and Supplementary Table 4).

The transformation with the *L. japonicus NIN* gene driven by the *NIN* minimal promoter (*NINmin*_*pro*_*:NIN*) did not alter the symbiotic phenotype of *nin-15* roots (Fig. 3b). By contrast, the *NIN* gene driven by the *NIN* promoter (*NIN*_*pro*_*:NIN*) led to restoration of the complete infection process in *nin-15* roots from root hair ITs to symbiosome formation (100% and 92% of transgenic root systems carried root hairs ITs and infected nodules, respectively; Fig. 3b). Similar to observations in complementation experiments of *nin-2*, mutation or deletion of *PACE* (*NIN*_*pro*_*::mPACE:NIN* and *NIN*_*pro*_*::∆PACE:NIN*, respectively) drastically reduced the restoration of bacterial infection in root hairs and nodules in *nin-15* (Fig. 3b, Extended Data Figs. 6–9 and Supplementary Figs. 6 and 7).

### *PACEs* from different nodulating FaFaCuRo species are functionally equivalent

*PACE* was detected by MEME searches as a conserved motif within *NIN* promoters of the FaFaCuRo clade. However, the individual *PACE* sequences from different species differed from each other, mostly so in the sequences flanking the *CYC-box* (Fig. 1 and Extended Data Fig. 1). We therefore tested whether and to what extend this sequence variation of *PACE* would affect its function. The replacement of *PACE* within the *L. japonicus* (Fabales) 3-kb *NIN* promoter with *PACE* sequence variants (*NIN*_*pro*_*::Species abbreviation PACE:NIN*) originating from *Casuarina glauca* (Fagales), *Datisca glomerata* (Cucurbitales) or *Dryas drummondii* (Rosales) restored the complete infection process in *nin-15* to similar level as *NIN*_*pro*_*:NIN*, demonstrating the functional conservation of *PACE* from nodulating species across the entire FaFaCuRo clade (Fig. 3c and Extended Data Fig. 9). Similarly, the *PACE* versions from two non-nodulating Rosales that maintained the *NIN* gene, *Ziziphus jujuba* and *Prunus persica*, restored the complete infection process in *nin-15* (Fig. 3d and Extended Data Fig. 9). The results of these complementation experiments were consistent with the conserved expression pattern mediated by *PACEs* in *L. japonicus* (Supplementary Fig. 8) and the CCaMK–Cyclops-mediated transactivation via these *PACE* variants (Extended Data Fig. 3a) or chimeric promoter:reporter fusions (Extended Data Fig. 3b) tested in *N. benthamiana* leaves.

### Loss of *PACE* is associated with a loss of the nitrogen-fixing RNS

Griesmann et al.^15^ and van Velzen et al.^16^ discovered that RNS was lost multiple times independently during evolution via independent truncations or losses of the *NIN* gene. However, at least 10 out of 28 FaFaCuRo species that lost RNS have maintained a full-length *NIN* open reading frame (Supplementary Table 3). On the basis of our complementation data, *PACE* is indispensable for the *NIN* promoter function in symbiosis (Fig. 3, Extended Data Figs. 6–9 and Supplementary Figs. 6 and 7). Therefore, the absence of *PACE* from five out of these ten species (Supplementary Tables 1–3), is potentially sufficient to explain these losses of RNS. Consequently, at least 82% of all losses can now be attributed to either the *NIN* ORF (18 out of 28, 64%) or loss of *PACE* (5 out of 28, 18%) (Supplementary Table 3). The presence of *PACE* in all nodulating species (31 out of 31, 100%; Supplementary Tables 1 and 2) together with a correlation between the absence of *PACE* with the absence of RNS adds strong support for the evolutionary relevance of *PACE* both in the gain and potential loss of RNS.

*PACE* was not detected in the promoters of *NLP* genes (Supplementary Fig. 1 and Supplementary Tables 1 and 2) with the possible exception of the curious case of *Juglans regia* (Fagales). Although it was also absent from the promoter of the so-annotated *NIN* gene, a *PACE*-like motif was identified in the promoter of the closest gene family member, *NLP1 JrNLP1b* (*JrPACE-like*; Supplementary Table 1). This *PACE*-like element was not able to restore IT formation in *nin-15* (Fig. 3d and Extended Data Fig. 9). Regardless of whether this exceptional presence/absence pattern of *PACE* may be caused by a miss-annotation of *NIN* and *NLP1* in *J. regia*, either a loss-of-function mutation within *PACE* or a loss of the entire *PACE* element in the *JrNIN* promoter could explain the absence of the RNS observed in this species.

### *PACE* is sufficient to restore cortical IT formation in *nin-15*

We tested whether *PACE* on its own, only supported by the minimal *NIN* promoter (*PACE:NINmin*_*pro*_), is sufficient to restore IT development in cortical cells. For this purpose, we transformed *nin-15* roots with *PACE:NINmin*_*pro*_ fused to the transcribed region of the *NIN* gene. *PACE*-mediated *NIN* expression led to an increased success in restoration of infection (49% of transgenic root systems carried infected nodules) compared with *NINmin*_*pro*_*:NIN*-transformed roots (17%; Fig. 4a, Extended Data Fig. 10 and Supplementary Table 4). Root hair ITs were rarely observed on *PACE:NINmin*_*pro*_*:NIN*-transformed *nin-15* roots (Fig. 4a), and nodules harbouring cells filled with symbiosomes were not found (Extended Data Fig. 10), consistent with the restricted expression domain defined by *PACE* (Extended Data Fig. 4).

Strikingly, the vast majority of infected nodules transformed with *PACE:NINmin*_*pro*_*:NIN* (25 out of 28 nodules inspected) carried ITs in the outer cortex, originating from a focused hyperaccumulation of bacteria, locally constricted by root cell wall boundaries (Fig. 4a). This phenomenon was not observed in most of the rarely occurring infected nodules formed on *NINmin*_*pro*_*:NIN*-transformed *nin-15* roots (11 out of the 16 nodules inspected did not carry ITs in the outer cortex). Bacterial colonies within cell wall boundaries resembling this phenomenon have been described in a variety of legumes including *Sesbania* and *Mimosa*^51,52^. Our data imply that *PACE* promotes this type of cortical IT initiation. Altogether, these findings revealed that *PACE* promotes IT development in cortex cells but not within root hairs.

### *PACE* insertion into the tomato *NIN* promoter confers RNS capability

To artificially recapitulate the functional consequence of *PACE* acquisition into a non-FaFaCuRo *NIN* promoter, we chose tomato (*Solanum lycopersicum*) which belongs to the Solanaceae, a family phylogenetically distant from the FaFaCuRo clade. Consistent with the absence of *PACE*, a *GUS* reporter gene driven by the tomato *NIN* promoter (*S. lycopersicum NIN* promoter (*SlNINpro*)) was not transactivated by Cyclops in *N. benthamiana* leaf cells (Fig. 1 and Extended Data Figs. 2b and 3c), whereas the insertion of the *L. japonicus PACE* (*SlNIN*_*pro*_*::PACE*), but not of a mutated *PACE* (*SlNIN*_*pro*_*::mPACE*), conferred transactivation by Cyclops (Extended Data Fig. 3c).

We tested the ability of the *LjNIN* expressed under the control of these synthetic promoters to restore the bacterial infection process in *nin-15*. Similar to *NINmin*_*pro*_*:NIN-*transformed *nin-15* roots, *SlNIN*_*pro*_*:NIN* did not restore bacterial infection (0% and 7% of transgenic root systems carried root hair ITs and infected nodules, respectively) (Fig. 4a–c). By contrast, *nin-15* roots transformed with *SlNIN*_*pro*_*::PACE:NIN* restored the formation of root hair ITs and infected nodules on 36% and 26% of transgenic root systems, respectively (Fig. 4b and Extended Data Fig. 10). This increase in infection success was not observed on *SlNIN*_*pro*_*::mPACE:NIN*-transformed roots. ITs in the outer cortex that originated from a focal accumulation of bacteria were also observed in the *SlNIN*_*pro*_*::PACE:NIN*-transformed *nin-15* nodules (8 out of 14 nodules inspected; Fig. 4b) resembling those in the *PACE:NINmin*_*pro*_*:NIN*-transformed *nin-15* nodules (Fig. 4a). The gained ability of the *SlNIN::PACE* promoter to restore root hair ITs suggested that additional *cis*-regulatory elements within the *SlNIN* promoter function together with *PACE* for root hair IT formation. All together, these findings obtained with the tomato *NIN* promoter carrying an artificially inserted *PACE* agree with the hypothesis that the acquisition of *PACE* by a non-FaFaCuRo *NIN* promoter enabled its regulation via Cyclops and laid the foundation for IT formation in cortical cells.

## Discussion

The mechanistic connection between *PACE* and cortical IT formation together with their congruent phylogenetic distribution strongly support the idea that the acquisition of *PACE* by the latest common ancestor of the FaFaCuRo clade enabled cortical ITs and thus laid the foundation for the evolution of present day RNS. Our findings support an evolutionary model in which an ancestral symbiotic transcription factor complex (comprising CCaMK and Cyclops), which facilitated intracellular symbiosis with AM fungi already in the earliest land plants^53,54^, gained control over the transcriptional regulation of the *NIN* gene by the acquisition of *PACE* (Fig. 1). This genetic innovation in the last common ancestor of the FaFaCuRo clade extended the function of the ancestral CCaMK complex to initiate cortical IT development. The *NLP* family underwent important evolutionary steps preceding the origin of RNS including a gene duplication leading to *NIN* and *NLP1* as closest paralogues^55^. It is very likely that the NIN protein itself underwent changes that enabled its role in nodulation^35^. Loss-of-*NIN* events associated with the loss of nodulation are scattered across all four FaFaCuRo orders^15,16^, suggesting that *NIN* acquired its relevance for nodulation probably before or latest in the last common ancestor. As our phylogenomic analysis dates the acquisition of *PACE* to the latest ancestor of the FaFaCuRo clade, we conclude that the critical changes within NIN must have occurred simultaneously or earlier. From a statistical point of view, it is likely that the *PACE* acquisition and the RNS-enabling changes within NIN occurred independently from each other. It will be interesting to determine what these critical changes within NIN are and where they occurred phylogenetically.

A ‘young’ primary cell wall characteristic for recently divided cells is considered an important prerequisite for cortical IT initiation^6,56^, but cell division is not restricted to the formation of novel organs^9^. It is therefore conceptually possible that the common ancestor of the FaFaCuRo clade was forming ITs in recently divided cortical cells but in the absence of root nodules. Multiple lines of evidence indicate that the diverse types of lateral organ harbouring nitrogen-fixing bacteria (‘nodules’) evolved multiple times independently. Indeed, *CE*-mediated *NIN* expression is important for nodule organogenesis in legumes, but upon searching for this regulatory element in a region of 0.1 Mb upstream and downstream of the *NIN* gene, Liu et al.^40^ found its presence to be restricted to legume species, indicating an evolutionary emergence independently of and considerably later than the last common ancestor^40,55^. ITs in root hairs are only found in Fabales and Fagales and therefore are also considered a more recent acquisition^6,57^. *CE* only in combination with *PACE* facilitates root hair ITs (Extended Data Figs. 6–8 and Supplementary Figs. 6 and 7) and additional elements in the 3 kb promoter are necessary for nodule and cortical IT development (Extended Data Figs. 6–8 and Supplementary Figs. 6 and 7). Furthermore, deletion of *PACE* by targeted genome editing was recently reported to reduce but not completely abolish the formation of root hair ITs, suggesting the presence of partly redundant *PACE* elements in the vicinity of the *LjNIN* gene^58^. Altogether, these observations highlight the complexity and concerted activity of *cis*-elements and transcription factors underlying the spatiotemporal expression control by present day *NIN* promoters in RNS-competent species. Our data pinpoint the acquisition of *PACE* as a key event during the evolution of the nitrogen-fixing RNS. Together with our discovery that multiple independent losses of *PACE* are associated with multiple losses of RNS within the FaFaCuRo clade, our data underpin the essential position of *PACE* in the evolutionary gain and loss of RNS.

## Methods

### Bioinformatic analyses

On the basis of the phylogenetic classification of the RWP-RK gene family^15,50^, 144 NIN/NLP genes were selected from 37 plant species and 13 orders ranging from monocotyledons to dicotyledons including the FaFaCuRo clade (Supplementary Table 1). For each selected gene, 3 kb of sequence upstream of the translational start site including the promoter and 5′ UTR region was defined and extracted from the corresponding species’ genomic sequence, if the contig length allowed it. For *Medicago truncatula*, a 3,352-bp sequence upstream of the translational start site was extracted. If contig length was limiting, the longest possible sequence stretch was extracted. For the identification of a *cis*-regulatory element specific for *NIN* promoters of the FaFaCuRo clade, the tool MEME^59^ was used in discriminative mode (‘search given strand only’ option, default parameters) with *NIN*-promoter regions of only nodulating plants. The control group consisted of promoter regions of all *NIN* genes outside of the FaFaCuRo clade and all *NLP* genes listed in Supplementary Table 1. The highest-scoring motif (*e* *=* 1.6 × 10^−58^) was 27-bp long and contained the much shorter previously described *CYC-box*^34^ (Extended Data Fig. 1a).

To refine the conserved region in this motif, MEME analysis was performed again in normal mode (‘search given strand only’ option, default parameters) with *NIN* promoters from only nodulating species. This analysis revealed that the most conserved nucleotides are found within 29 nucleotides (nucleotides 10 to 38 in Extended Data Fig. 1b). The previous MEME analysis was repeated, but an exactly 29-nucleotide-long motif was searched for (resulting in a motif in Extended Data Fig. 1c), and the best-scoring *NIN* paralogue per searched species, that is, the lowest *P*value per species, were identified (Supplementary Table 1). In a final step, one best-scoring *NIN*-promoter region per nodulating species were analysed with MEME (‘search given strand only’ option, default parameters) by searching for an exactly 29-nucleotide-long motif. The resulting motif was named *PACE* (Extended Data Fig. 1d). This final MEME run was done for two reasons: first, to avoid a sequence bias towards a single species with multiple *NIN* paralogue promoter regions (for example, soybean) and, second, to avoid a potential sequence bias generated by the promoter region of a *NIN* paralogue that might be no longer functional and therefore has mutated sites in its promoter region owing to relaxed selection pressure.

As a control, the FIMO^59^ tool was used (‘scan given strand only’ option, default parameters, false discovery rate < 0.1) to search all 144 *NIN* and *NLP* promoter regions (Supplementary Table 1). *PACE* was found within *NIN*-promoter regions of all nodulating species analysed and two non-nodulating FaFaCuRo species (*Prunus persica* and *Ziziphus jujuba*) (Supplementary Table 1).

The presence or absence of *PACE* was further investigated in promoters of *NIN* and *NLPs* in an expanded database of 163 species encompassing 39 orders covering six groups of Viridiplantae (Supplementary Table 2). Orthologues of the whole *NLP* family were retrieved using tBLASTn v2.11.0+^60^ with reference sequences from *Medicago truncatula* as query and a cut-off *e*value of 1 × 10^−10^. Sequences were then aligned using MAFFT v7.380^61^ with default parameters. To identify the NIN and NLPs orthologues and therefore resolve the NIN and NLP protein subfamilies, we used a maximum likelihood approach using the IQ-TREE v1.6.7 software^62^. Before phylogenetic reconstruction, the best-fitting evolution model was determined for each alignment using ModelFinder^63^ as implemented in IQ-TREE. Branch support was tested using 10,000 replicates of UltraFast Bootstraps using UFBoot2^64^. For each identified orthologue, a 5-kb region upstream of the translational start site was extracted. The three different consensuses identified in the previous MEME analyses (Extended Data Fig. 1a,b,d) were then searched in all *NLP* upstream regions using FIMO 5.0.2 and a *q*-value threshold of 0.1. If several motifs were identified in a given upstream region, only the one with the lowest *q*-value was conserved for further analysis (Extended Data Fig. 1e, Supplementary Fig. 1 and Supplementary Table 2). *PACE* from *Parasponia andersonii* (a nodulating species from Rosales) was identified in this analysis and included to generate the consensus in Fig. 1.

The promoters of *ERN1* and *RAM1* genes were analysed independently of the previous analysis, using 87 plant genomes covering the main Angiosperms orders (Supplementary Table 5). Orthologues of each gene were retrieved using tBLASTn v2.7.1+^60^ with reference sequences from *Medicago truncatula* as query and a cut-off *e*-value of 1 × 10^−10^. The sequences were then aligned using MAFFT v7.380^61^ with default parameters. The alignments were subjected to phylogenetic analysis to identify orthologs using maximum likelihood approach and the IQ-TREE v1.6.7 software^62^. Before phylogenetic reconstruction, the best-fitting evolution model was determined for each alignment using ModelFinder^63^ as implemented in IQ-TREE. The branch support was tested using 10,000 replicates of UltraFast Bootstraps using UFBoot2^64^. For each identified orthologue, the regions upstream of the translational start site of different lengths (1 to 5 kb) were extracted. For each length of region upstream of the translational start site, the sequences were analysed using MEME software v5.0.1(ref. ^59^) with the following parameters: a motif size between 5 and 45 bp and 5 and 25 bp for *ERN1* and *RAM1*, respectively, and a maximum number of discovered motifs of 20 (Supplementary Figs. 2 and 3). In addition, MEME search was set on ‘zoops’ mode, assuming that each sequence can contain zero or one occurrence of the motif.

### Biological material

*L. japonicus* ecotype Gifu B-129 wild-type^65^, *nin-2*^32^ and *nin-15* (*LORE1* line 30003529^66^) were used in this study. Seed bags, bacterial strains and days post inoculation for each experiment are listed in Supplementary Table 6.

### Plant growth conditions and symbiotic inoculations

*Lotus japonicus* seeds were scarified and surface-sterilized as described^67^ before germination on ½ Gamborg’s B5 medium solidified with 0.8% Bacto agar in square plates (12 cm ×12 cm × 1.7 cm)^68^. The lates were kept in dark for 3 days before transferring to light condition in a Panasonic growth cabinet (MLR-352H-PE) at 24 °C under a 16 h–8 h light–dark regime (50 µmol m^−2 ^s^−1^). The 6-day-old seedlings were (1) subject to hairy root transformation as described^69^ (Figs. 2–4, Extended Data Figs. 4–10 and Supplementary Figs. 5–8) or (2) transferred to Weck jars (SKU 745 or 743; J.Weck GmbH u. Co. KG) containing 300 ml of sand:vermiculite mixture (2:1) and 20 ml of a modified ¼ strength Hoagland’s medium with Fe-EDDHA used as iron source^70^ (Extended Data Fig. 5e). For in vivo promoter expression analysis (Fig. 2 and Supplementary Fig. 5), transgenic roots expressing a kanamycin-resistance gene were kept on square plates supplemented with kanamycin (25 µg ml^−1^) 10 days after the *Agrobacterium rhizogenes* inoculation. Plants with transformed roots were kept on 0.8% Bacto agar including a nitrogen-reduced version of FAB medium (500 µM MgSO_4_·7H_2_O, 250 µM KH_2_PO_4_, 250 µM KCl, 250 µM CaCl_2_·2H_2_O, 100 µM KNO_3_, 25 µM Fe-EDDHA, 50 µM H_3_BO_3_, 25 µM MnSO_4_·H_2_O, 10 µM ZnSO_4_·7H_2_O, 0.5 µM Na_2_MoO_4_·2H_2_O, 0.2 µM CuSO_4_·5H_2_O, 0.2 µM CoCl_2_·6H_2_O; pH 5.7) in square plates for 1 week before transferring to a growth chamber at 24 °C under a 16 h–8 h light–dark regime (275 µmol m^−2 ^s^−1^) in Weck jars (SKU 745 or 743) containing 300 ml of sand:vermiculite mixture (2:1) and 30 ml of nitrogen-reduced FAB medium containing *Mesorhizobium loti* MAFF 303099 *Ds*Red^71^ (*M. loti Ds*Red; Figs. 3 and 4, Extended Data Figs. 5–10 and Supplementary Figs. 6 and 7), *M. loti* R7A CFP^72^ (Fig. 2) or M. *loti* MAFF 303099 GFP^73^ (Supplementary Fig. 5) set to a final optical density at 600 nm (OD_600_) of 0.05. For Extended Data Fig. 4 and Supplementary Fig. 8, plants were grown in Weck jars (SKU 745 or 743) containing 300 ml of sand:vermiculite mixture (2:1) and 60 ml of nitrogen-reduced FAB medium containing *M. loti Ds*Red or MAFF 303099 *lacZ*^72^ (*M. loti lacZ*) (OD_600_of 0.01).

### Cloning and DNA constructs

For the construction of promoter:*NIN* fusions for complementation experiments (Fig. 3, Extended Data Figs. 6–10 and Supplementary Figs. 6and 7), the *NIN* genomic sequence without the 5′ and 3′ UTRs served as a cloning module. A 3-kb region of the *L. japonicus NIN* promoter plus the 244 bp *NIN* 5′ UTR was cloned from *L. japonicus* Gifu and used for complementation experiments (Fig. 3, Extended Data Figs. 6–9 and Supplementary Figs. 6 and 7), dual-luciferase assays (Extended Data Fig. 2), fluorimetric GUS assay (Extended Data Fig. 3) and promoter activity analysis (Fig. 2, Extended Data Fig. 4 and Supplementary Fig. 5). For all the other versions of the *L. japonicus NIN* promoter tested (Figs. 2–4, Extended Data Figs. 3–10 and Supplementary Figs. 5–8), the *LjNIN* minimal promoter (98 bp)^34^ plus the *LjNIN* 5′ UTR was fused to the 3′ end of the promoter. A 472-bp region containing multiple cytokinin response elements and highly conserved in eight legume species was identified 5′ of the *NIN* transcriptional start site by Liu et al.^40^. We used this conserved region of 472 bp from *L. japonicus* and added flanking regions (192 bp upstream and 366 bp downstream and 2,399 bp upstream and 2,231 bp downstream, respectively) to obtain cytokinin element-containing regions of 1 kb and 5 kb (*CE*_*1kb*_ and *CE*_*5kb*_, respectively). The *S. lycopersicum* gene ID Solyc01g112190.2.1 was identified as the closest homologue of *LjNIN* gene on the basis of phylogenetic analysis^15^ and is referred to as *SlNIN*. A 3-kb region of the *SlNIN* promoter plus the 238-bp *SlNIN* 5′ UTR was cloned from *S. lycopersicum* cv. ‘Moneymaker’ and *PACE* or *mPACE* (Extended Data Fig. 3a) was inserted 184 bp upstream of the *SlNIN* 5′ UTR and used for complementation experiments (Fig. 4 and Extended Data Fig. 10), dual-luciferase assays (Extended Data Fig. 2) and fluorimetric GUS assay (Extended Data Fig. 3). A detailed description of constructs can be found in Supplementary Fig. 4 and Supplementary Table 7. A list of oligonucleotides can be found in Supplementary Table 8. The constructs were generated with the Golden Gate cloning system^74^.

### Imaging

Microscope and scanner settings as well as parameters for image acquisition are listed in Supplementary Table 9.

### Phenotypic analysis and quantification of infection events

Infected and non-infected nodules were discriminated by the presence and absence of a *Ds*Red signal (representing *M. loti Ds*Red) detected or not detected inside of the nodules, respectively. The presence or absence of bacteria was later confirmed by examination of sections of representative nodules. ITs and *M. loti* entrapments in root hairs were detected by their *Ds*Red fluorescence (see Supplementary Table 9 for microscope settings).

For the phenotypic analysis of *nin-15* (Extended Data Fig. 5c,f), quantification was performed 21 dpi with *M. loti Ds*Red as follows: (1) the total number of nodules (including infected and non-infected) was determined under white light illumination, and (2) the number of infected nodules and root hair ITs were counted as described above. Shoot dry weight was measured after drying the shoot at 60 °C for 1 h (Extended Data Fig. 5e).

For the complementation experiments of *nin-2* and *nin-15* (Figs. 3 and 4, Extended Data Figs. 6–10 and Supplementary Figs. 6 and 7), quantifications and sectioning were performed 21 or 35 dpi with *M. loti Ds*Red with the microscope settings listed in Supplementary Table 9 in the following order: (1) transgenic roots were identified by GFP fluorescence-emanating nuclei with a GFP filter, (2) infected nodules were counted as described above, (3) the total number of nodules (including infected and non-infected ones) was then determined under white light illumination and (4) the number of non-infected nodules was calculated by subtracting the number of infected nodules from the total number of nodules. To quantify infection events in root hairs, the number of bacterial entrapment and ITs in root hairs were counted on a 0.5-cm root piece for each transgenic root system, excised from a region where bacterial accumulation was detected by *Ds*Red fluorescence. Sectioning was performed on non-infected and infected nodules, and the presence/absence of ITs and symbiosomes in cortical cells was examined. Nodule primordia and nodules were embedded in 6% low-melting agarose and sliced into 40–50-µm thick sections using a vibrating-blade microtome (Leica VT1000 S).

### Transient expression in *Nicotiana benthamiana* leaves

*Agrobacterium tumefaciens* strain AGL1 carrying promoter:reporter fusions on T-DNA were infiltrated as previously described^75^ with the acetosyringone concentration in the infiltration buffer modified to 150 µM. *A. tumefaciens* strains AGL1 and GV3101 containing plasmids *35S*_*pro*_*:3xHA-Cyclops*^34^ and *35S*_*pro*_*:CCaMK*^*1–314*^*-mOrange*^76^, respectively, were co-infiltrated with the reporter constructs as indicated. An AGL1 strain carrying a K9 plasmid constitutively expressing red fluorescent protein was used as needed to equalize the density of the *A. tumefaciens* suspension infiltrated per leaf, together with an *A. tumefaciens* strain carrying a plasmid for the expression of the viral P19 silencing suppressor to reduce post-transcriptional gene silencing^77^ (Extended Data Figs. 2 and 3). *N. benthamiana* leaf discs with a diameter of 0.5 cm were harvested 60 h post infiltration and used for quantitative fluorometric GUS assay and dual-luciferase assay.

### Dual-luciferase assay

The dual-luciferase assay (Extended Data Fig. 2) was based on the Dual-Luciferase reporter assay system (Promega). *N. benthamiana* leaf discs were ground to a fine powder in liquid nitrogen in 2 ml Eppendorf Safe-Lock tube (one leaf disc in each tube) and subsequently incubated for 5 min at room temperature with 200 µl of the Passive lysis buffer (Promega E1910). The resulting crude leaf extract was centrifuged at 20,000*g* for 2 min at room temperature. An aliquot of the supernatant was subjected to the dual-luciferase assay according to manufacturer’s instruction for Promega Dual-Luciferase Kit (E1910) and chemiluminescence was quantified with a fluorescence plate reader (TECAN Infinite 200 PRO; TECAN Group) in white 96-well plates (Greiner Bio-One International). For each reporter construct, the promoter of interest was fused to the *Firefly* luciferase gene, and constitutively expressed *Renilla* luciferase from the same vector was used for normalization (Supplementary Table 7). The ratio of the two signals (*Firefly* luciferase signal to the *Renilla* luciferase) was calculated and normalized to the vector control. A total number of at least four biological and two technical replicates per indicated vector were analysed in two independently performed assays.

### Quantitative fluorometric GUS assay and analysis

Quantitative fluorometric GUS assays (Extended Data Fig. 3) were performed as described^78^ adapted to the 96-well format. A total number of seven to eight leaf discs per indicated vector combination were analysed in two assays independently performed in different weeks.

### Promoter activity analysis

For promoter activity analyses with the *GUS* reporter gene (Extended Data Fig. 4a–f and Supplementary Fig. 8), transgenic nodule primordia and nodules were excised 10–14 dpi or ≥21 dpi with *M. loti Ds*Red and stained for GUS activity using 5-bromo-4-chloro-3-indolyl-β-d-glucuronic acid (X-Gluc; x-gluc.com) as catalytic substrate^75^ for 3 h at 37 °C. To visualize the root hair ITs together with the promoter activity with the *GUS* reporter gene (Extended Data Fig. 4g), plants with transgenic root systems were inoculated with *M. loti lacZ*. Transgenic roots were first stained for GUS activity with X-Gluc for 3 h at 37 °C and then for *lacZ* expression with Magenta-Gal for 18 h at 28 °C (as described in ref. ^75^, which were visualized in blue and purple colours after staining, respectively). For promoter activity analyses with fluorescent reporters (Fig. 2 and Supplementary Fig. 5), transgenic root systems were harvested 7 dpi (Supplementary Fig. 5) or 10–14 dpi (Fig. 2). Roots with bacterial infection at stage 2 or 3 and nodule primordia with bacterial infection at stage 3 or 4 (see main text for stage description) were selected by locating the GFP (*M. loti* MAFF 303099 GFP) and CFP signal (*M. loti* R7A CFP), respectively, via rapid (around 10 s) *Z*-stack analysis with the confocal light scanning microscope (Supplementary Table 9). For cell wall staining with calcofluor white (Supplementary Fig. 5), roots were fixed with 4% formaldehyde dissolved in 50 mM piperazine-*N*,*N*′-bis(2-ethanesulphonic acid) (PIPES) buffer (pH 7) for 1 h under vacuum, rinsed three times with PIPES buffer and incubated in 0.05% calcofluor white dissolved in H_2_O for 1 h. The roots and sections of nodule primordia were imaged as described in Supplementary Table 9.

### Data visualization and statistical analysis

Statistical analyses and data visualization were performed with RStudio 1.1. 383 (RStudio Inc.). Box plots were used to display data in Fig. 4, Extended Data Figs. 2, 3 and 5–10 and Supplementary Fig. 6 (thick black or white lines, median; box, interquartile range (IQR); whiskers, lowest and highest data point within 1.5× IQR; black-filled circles, data points inside 1.5× IQR; white-filled circles, data points outside 1.5× IQR of the upper/lower quartile). The R package beeswarm with the method ‘center’ was used to plot the individual data points for the box plots^79^. The R package agricolae was used to perform ANOVA statistical analysis with post hoc Tukey, and statistical results are displayed in small letters where different letters indicate statistical significance^80^. The tests applied are stated in the figure legends.

### Reporting summary

Further information on research design is available in the Nature Portfolio Reporting Summary linked to this article.

## Supplementary information

Supplementary Information Supplementary Figs. 1–8.

Reporting summary

Supplementary Table 1 Summary of the bioinformatic analysis resulting in the discovery of *PACE* using 37 species.

Supplementary Table 2 Results of the FIMO analysis of *PACE* in 163 species.

Supplementary Table 3 Status of *PACE* and *NIN* in non-nodulating FaFaCuRo species. The species highlighted in grey are non-nodulating FaFaCuRo species that possess a full-length *NIN* open reading frame.

Supplementary Table 4 Results of hairy root mediated complementation experiments of the *L. japonicus* Gifu *nin-2* and *nin-15* mutant lines with indicated constructs.

Supplementary Table 5 List of plant genomes used for the search of conserved motifs within *ERN1* and *RAM1* promoters.

Supplementary Table 6 List of seed bags, bacterial strains and incubation times.

Supplementary Table 7 List of plasmids used.

Supplementary Table 8 Sequences and IDs of oligonucleotides (DNA) used.

Supplementary Table 9 Microscope/scanner settings and image analysis.

## Data Availability

Raw data corresponding to Fig. 1, Extended Data Fig. 1 and Supplementary Figs. 1–3 are available in Supplementary Tables 1–3 and 5. The remaining raw data are available on Zenodo (10.5281/zenodo.20719943). Essential plasmids listed in Supplementary Table 7 can be ordered from the European Plasmid Repository (https://www.plasmids.eu/). References for the *L. japonicus* lines and *M. loti* strains are indicated in Supplementary Table 6.
