# Supplementary material for: A novel cis-element enabled bacterial uptake by plant cells
Source: Nat Plants. 2026 Jan 2;12(1):140–51. doi: 10.1038/s41477-025-02161-z (PMC12830364; doi:10.1038/s41477-025-02161-z)
Supplement: Supplementary file 1 — Supplementary Figs. 1–8. [file 41477_2025_2161_MOESM1_ESM.pdf]

nature plants

Supplementary information

https://doi.org/10.1038/s41477-025-02161-z

A novel *cis*-element enabled bacterial uptake by plant cells

In the format provided by the authors and unedited

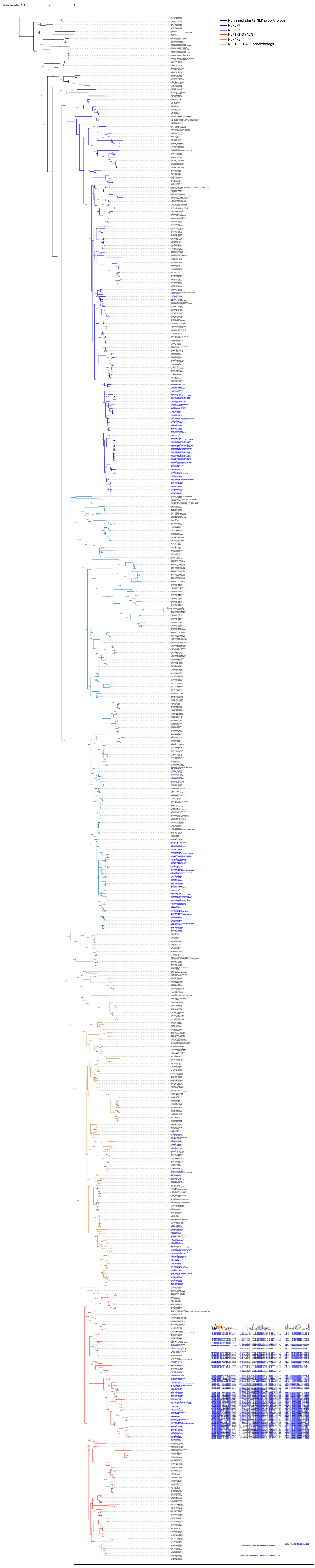

Supplementary Figure 1 | *PACE* is present exclusively in the FaFaCuRo clade. Motif analyses by FIMO using the using the regions upstream of the translational start site (ATG) thus representing the promoters and 5'UTRs of the *NIN* and *NLP* genes from an expanded list of 163 species (see Methods). Left: Maximum likelihood tree of *NLP* family (model: GTR+F+R10) of 163 species (**Supplementary Table 2**). Tree was rooted on the Charophytes algae *Klebsormidium nitens*. Black box demarcates the area that is displayed in **Extended Data Fig. 1E**. Blank lines represent the absence of significant motifs. Sequence names of nodulating species are coloured in blue. The different NLP clades are indicated by branch colours and named according to the *Arabidopsis thaliana* nomenclature. Right: three versions of consensus sequence (left to right, **Extended Data Fig. 1A, 1B and 1D**, respectively) were used to retrieve motifs from the 5 kb upstream region of *NIN* genes via FIMO search, the output of which is displayed underneath the consensus sequences.

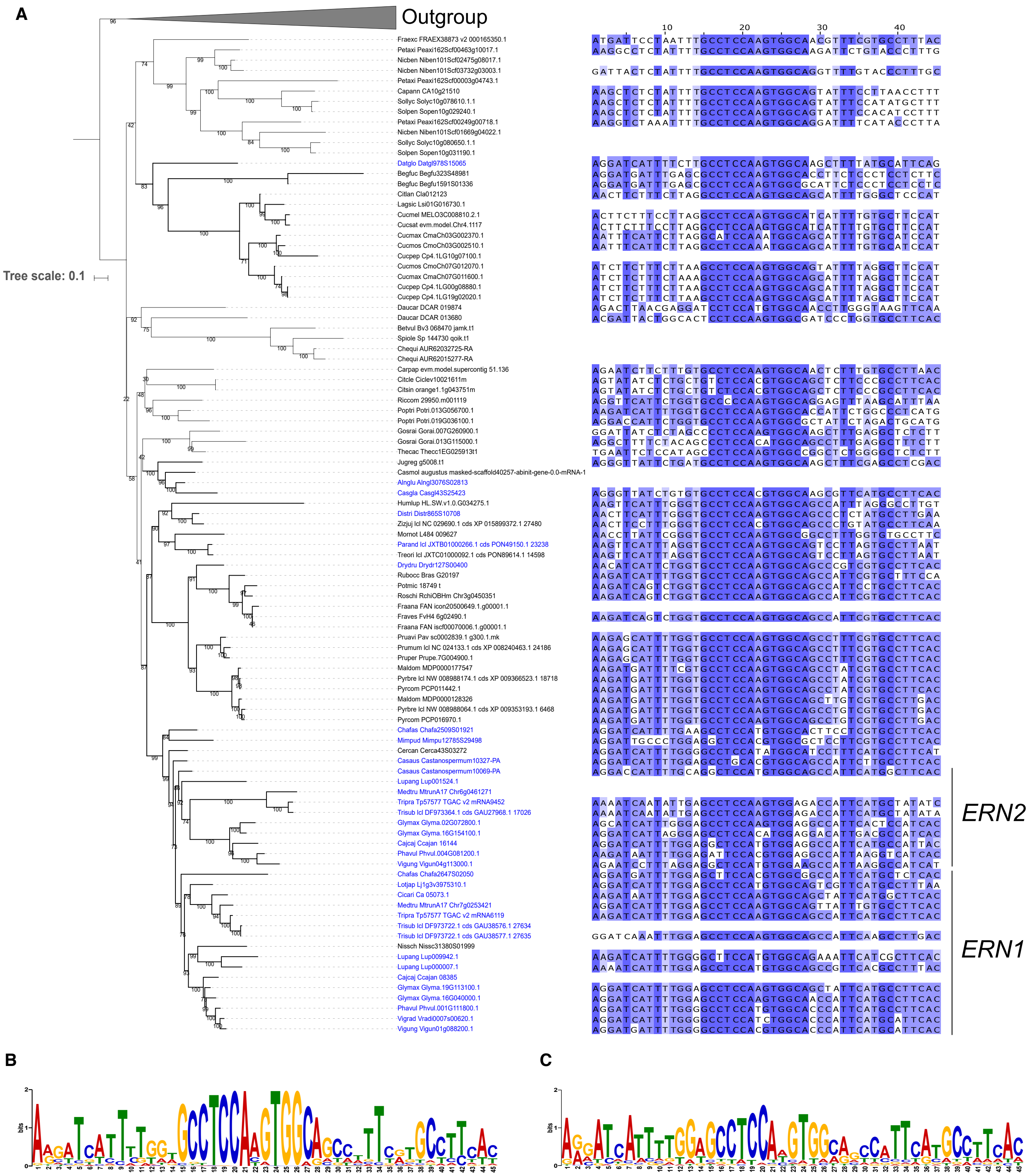

Supplementary Figure 2 | The presence of a motif encompassing the previously identified Cyclops binding site within the regions upstream of the translational start site (ATG) thus representing the promoters and 5'UTRs of *ERN1* genes extends beyond the FaFaCuRo clade. (**A**) Maximum likelihood of *ERN1/ERN2* (model: JTT+F+R5) from 87 species (**Supplementary Table 5**) based on protein sequences. Tree was rooted using sequences from the *ERN1/ERN2* paralog *ERN3* (outgroup clade collapse and indicated by grey triangle marked "Outgroup"). Species belonging to the monophyletic FaFaCuRo clade (Stevens, http://www.mobot.org/MOBOT/research/APweb/) are indicated by thicker black branches. *ERN1* and *ERN2* subclades are indicated. Sequence names of nodulating species are coloured in blue. These conserved motifs identified by MEME analyses in the 3 kb upstream regions were aligned and displayed next to species names. While the presence of a conserved Cyclops binding site containing motif in species outside of the FaFaCuRo clade is consistent with the role of *RAM1* in arbuscular mycorrhizal symbiosis, a function of *ERN1* in arbuscular mycorrhizal symbiosis has not been described and suggests a yet unidentified function of *ERN1* outside of the FaFaCuRo clade. (**B**) Consensus sequence of the Position Weight Matrix identified by MEME analyses of all the species. (**C**) Consensus sequence of the Position Weight Matrix identified by MEME analyses when analysing only the nodulating species.

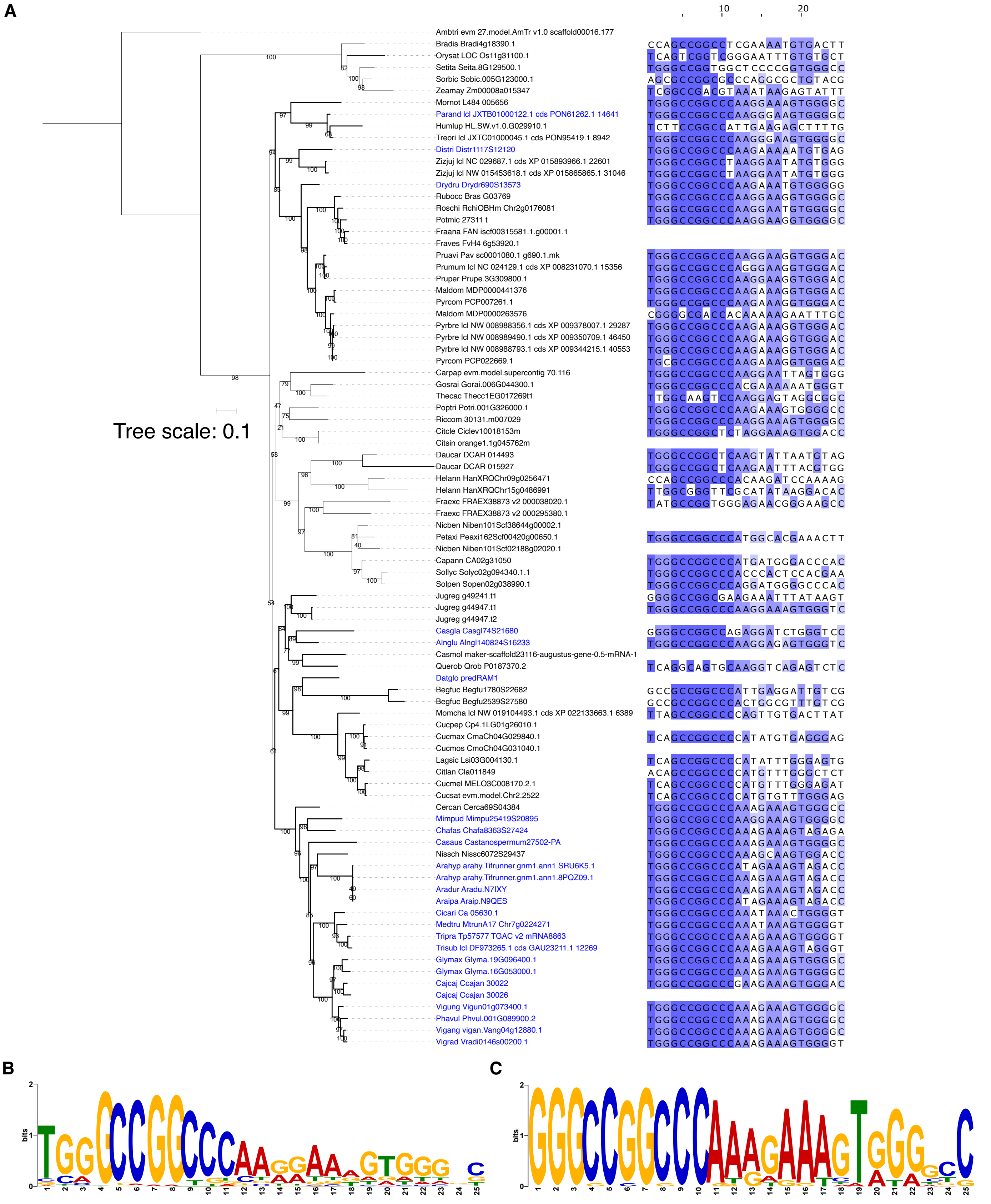

Supplementary Figure 3 I The presence of a motif encompassing the previously identified Cyclops binding site within the regions upstream of the translational start site (ATG) thus representing the promoters and 5'UTRs of *RAM1* genes extends beyond the FaFaCuRo clade. (**A**) Maximum likelihood of *RAM1* (model: JTT+F+R4) from 87 species (**Supplementary Table 5**) engaging in arbuscular mycorrhizal symbiosis. Tree was rooted on the early-diverging angiosperm *Amborella trichopoda*. Species belonging to the monophyletic FaFaCuRo clade (Stevens, http://www.mobot.org/MOBOT/research/APweb/) are indicated by thicker black branches. Sequence names of nodulating species are coloured in blue. These conserved motifs identified by MEME analyses in the 3 kb upstream regions are aligned and displayed next to species names. Blank lines represent the absence of identified motif due to genome contiguity issues. (**B**) Consensus sequence of the Position Weight Matrix identified by MEME analyses when analysing all the mycorrhizal species. (**C**) Consensus sequence of the Position Weight Matrix identified by MEME analyses when analysing only the nodulating species.

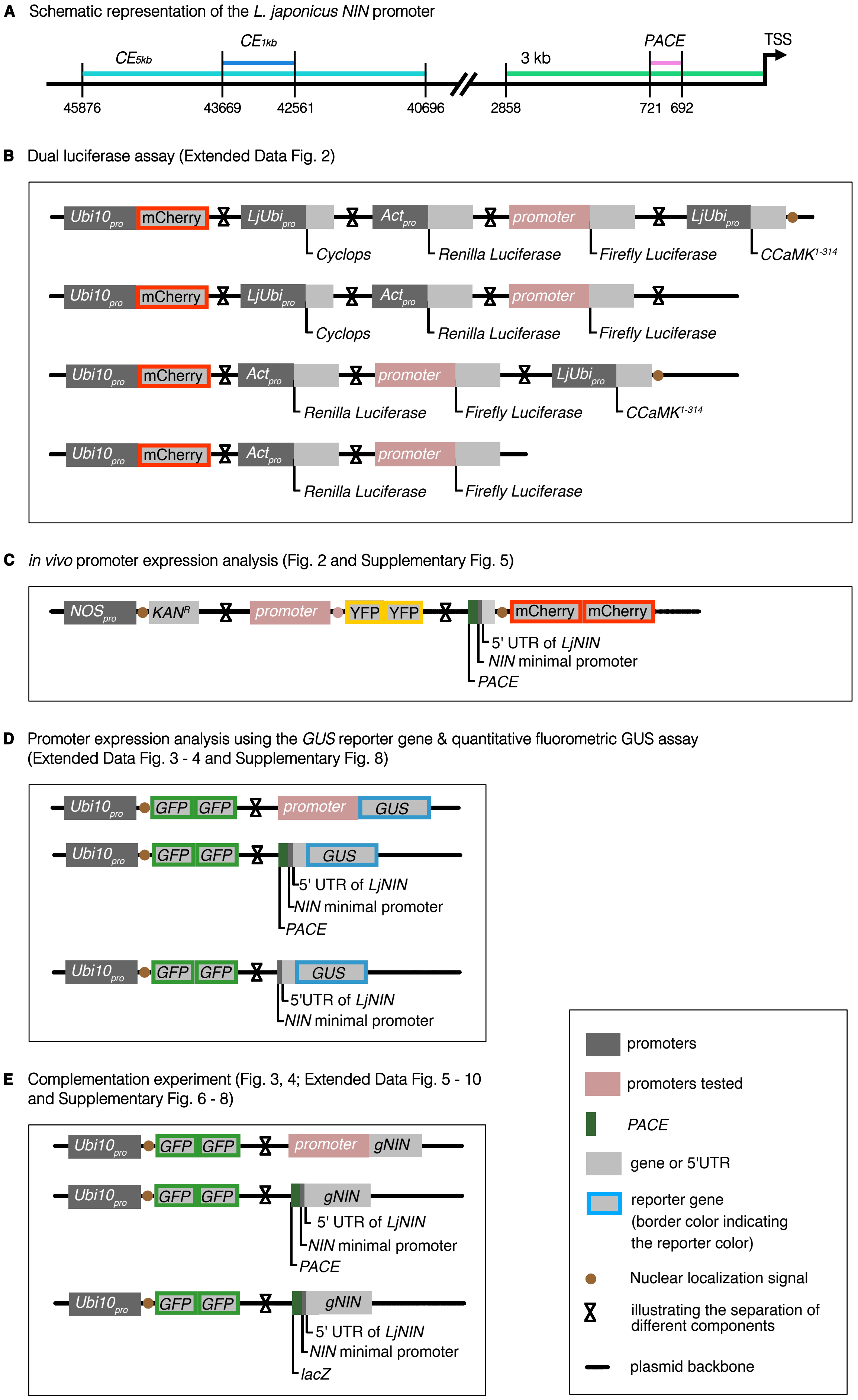

Supplementary Figure 4 I Schematic representation of the *NIN* promoter and T-DNA constructs used in this study. (**A**) Schematic representation of the *L. japonicus NIN* promoter and *cis*-regulatory regions that control *NIN* expression and enable rhizobia infection and nodule development. The CE_{1kb} and CE_{5kb} regions encompass several putative cytokinin response elements[40]. The 3 kb long promoter region encompasses *PACE* as well as the NSP1 and IPN2 binding sites[47,48]. Numbers indicate the number of bases from the transcriptional start site (TSS). (**B - E**) Schematic drawing of the T-DNA region of plasmids used in different experiments. Details of these plasmids are listed in **Supplementary Table 7**.

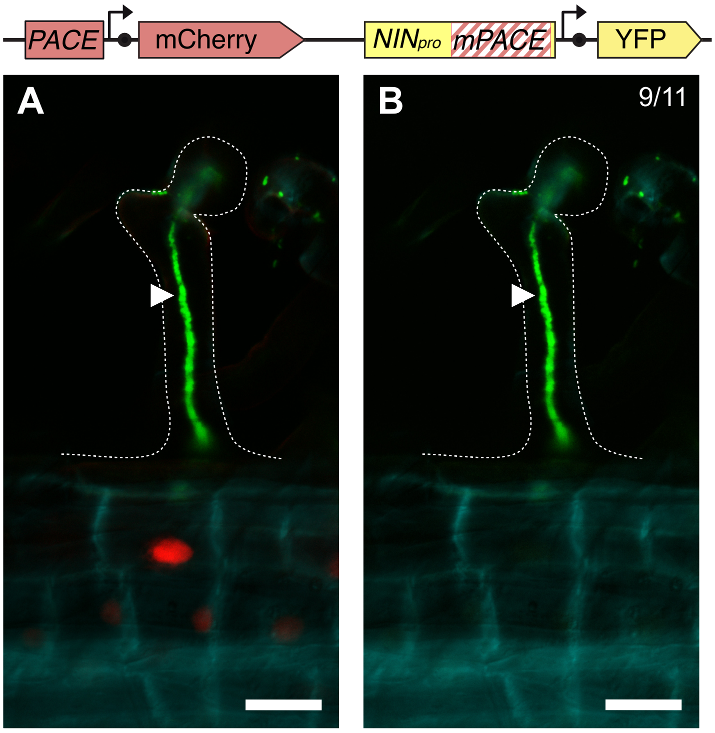

Supplementary Figure 5 | *PACE* drives the expression of the mCherry fluorescent reporter in cortical cells during IT development. Representative pictures of *L. japonicus* root upon inoculation with *M. loti* MAFF 303099 expressing GFP (green) imaged by confocal laser-scanning microscopy. Comparison of the expression domains determined by (**A**) *PACE* (*PACE:NINmin$_{pro}$:NLS-mCherry*; red) and (**B**) a *NIN* promoter carrying a mutated *PACE* (*NIN$_{pro}$::mPACE:NLS-YFP*; yellow). The plant cell wall was stained with calcofluor white (cyan). Dashed lines indicate the membrane of the root hair cell. Arrowheads indicate the IT. Numbers: roots showing the presented expression pattern / total number of roots inspected. Data are from a single experiment. Bars, 20 μm.

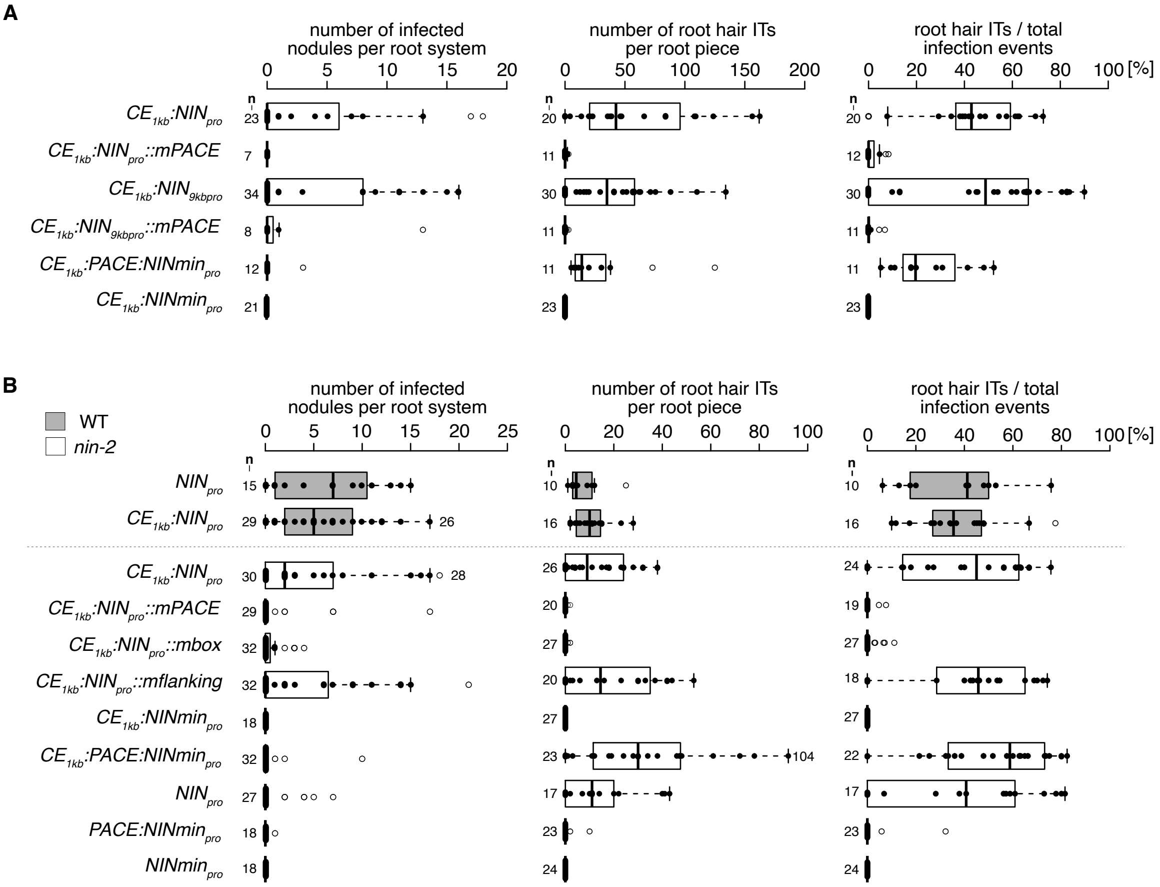

Supplementary Figure 6 | The *CYC-box* and flanking sequences of *PACE* are required for the complete restoration of the bacterial infection process in the *L. japonicus nin-2* mutant. Roots were from a subset of plants from the same experiment depicted in **Extended Data Fig. 6** but analysed 35 dpi with *M. loti Ds*Red. (**A - B**) Boxplots displaying the number of root hair ITs or infected nodules and the percentage of root hair ITs among total infection events (sum of bacterial entrapments and ITs). Each dot represents one transgenic *nin-2* root system or root piece. *L. japonicus* WT roots transformed with NIN_{pro}:*NIN* or CE_{1kb}:NIN_{pro}:*NIN* were included as controls. Note that the results follow the same trend as those obtained 21 dpi with *M. loti Ds*Red (**Extended Data Fig. 6**). n: number of transgenic root systems or root pieces analysed. Thick black lines, median; box, interquartile range; whiskers, lowest and highest data point within 1.5 interquartile range (IQR); black filled circles, data points inside 1.5 IQR; white filled circles, data points outside 1.5 IQR of the upper/lower quartile. Numbers above the boxplots: the value of individual data points outside of the plotting area. Data are from a single experiment.

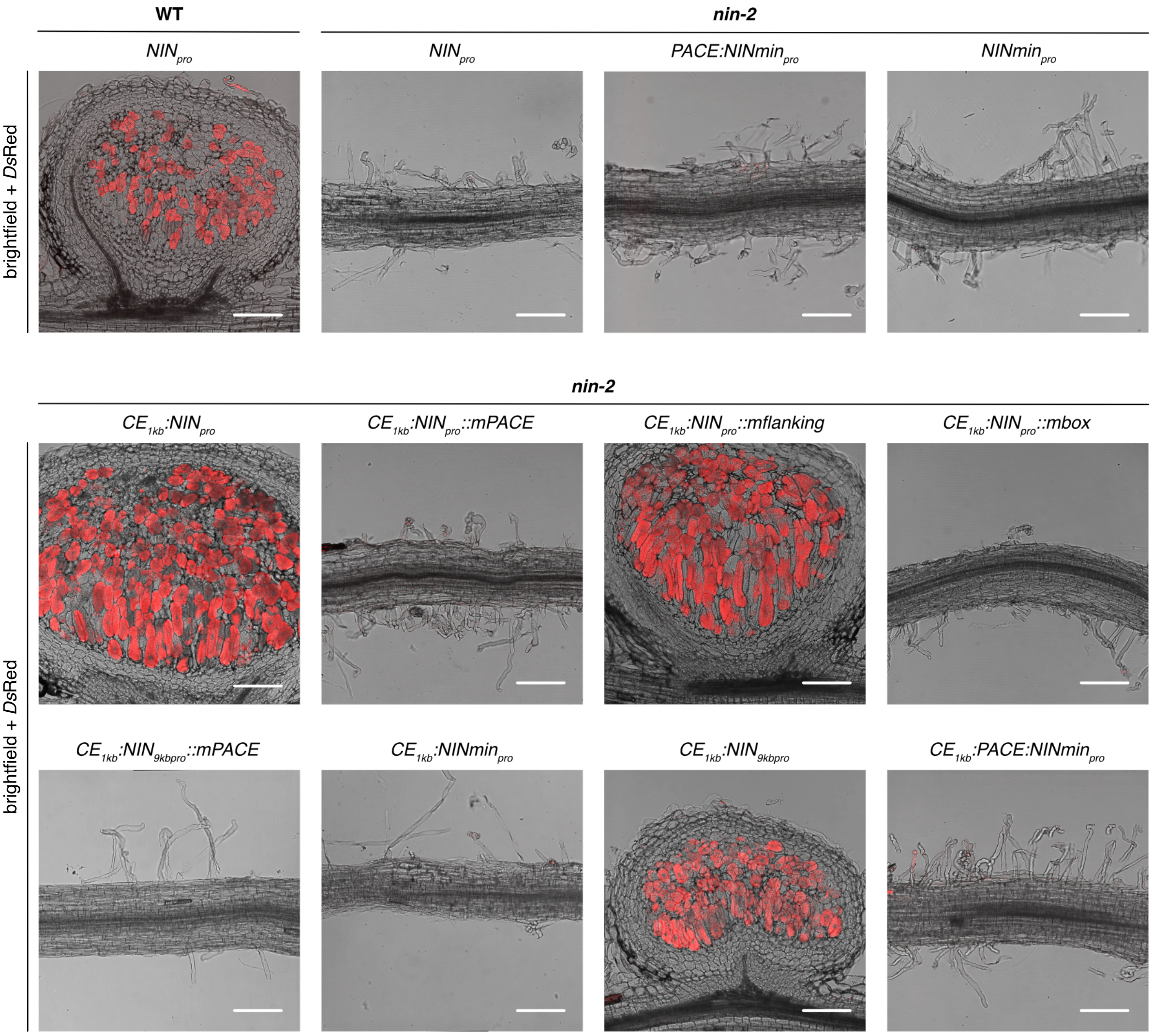

Supplementary Figure 7 | The *CYC-box* and flanking sequences of *PACE* are required for the complete restoration of the bacterial infection process in the *L. japonicus nin-2* mutant. Pictures of nodule sections or roots from *L. japonicus nin-2* roots 35 dpi with *M. loti Ds*Red from the same experiments depicted in **Supplementary Fig. 6**. Upper left corner: a nodule section from a *L. japonicus* WT root transformed with NIN_{pro}*:NIN* was included for comparison. At least five nodules from independent transgenic root systems were sectioned per construct. Bars, 100 µm.

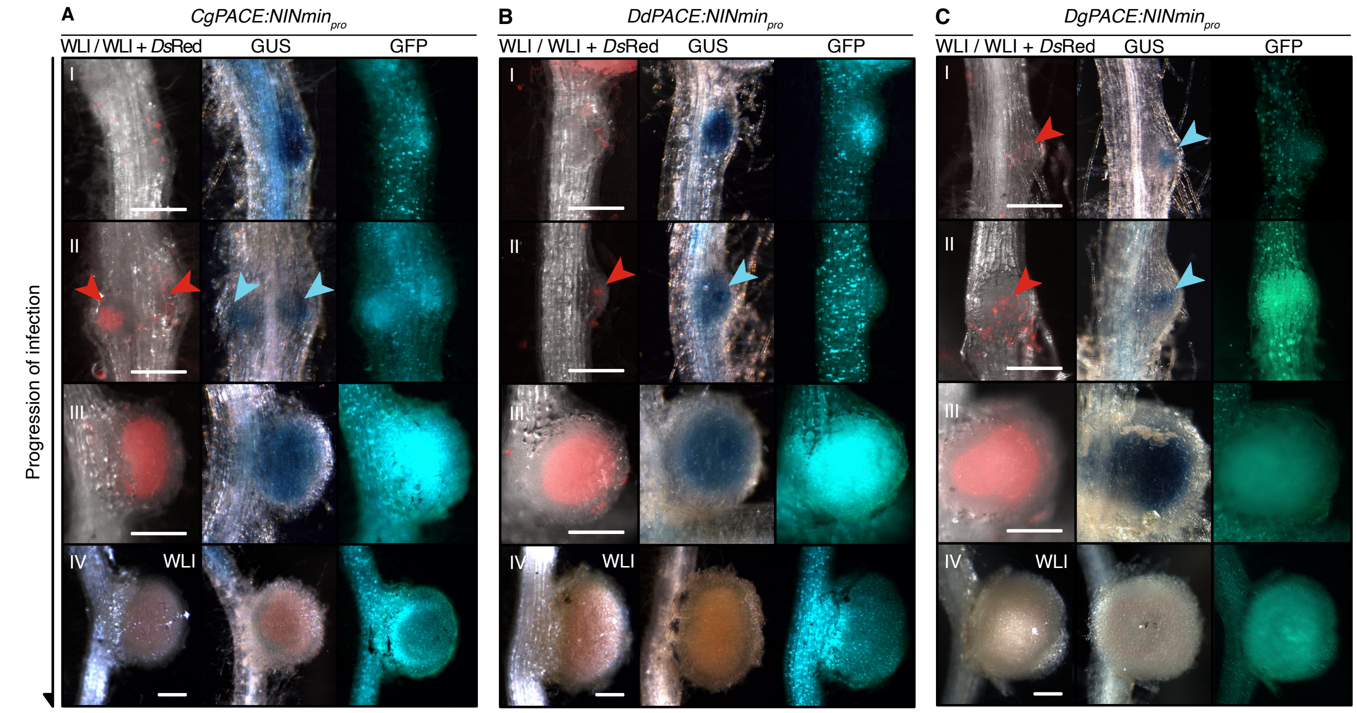

D

promoter:GUS	Time point (dpi)		
	0	10 -14	≥ 21
$CgPACE:NINmin_{pro}$	7[0]/7	14[23]/19	15[2]/20
$DdPACE:NINmin_{pro}$	8[0]/8	15[23]/18	17[2]/22
$DgPACE:NINmin_{pro}$	n.d.	35[23]/46	28[2]/38

#/#: root systems exhibiting GUS activity / total root systems analysed
[0] vasculature (of roots or nodules) and/or root tips
[1] epidermis (including root hairs)
[2] nodule primordia
[3] central tissue of nodules

Supplementary Figure 8 l Spatio-temporal *GUS* expression driven by *PACE* variants in *L. japonicus* roots during the bacterial infection process. *L. japonicus* WT roots were transformed with T-DNAs carrying a $Ubq10_{pro}$*:NLS-GFP* transformation marker together with a *GUS* reporter gene driven by either of the *PACE* variants from nodulating FaFaCuRo species fused to the *LjNIN* minimal promoter ($NINmin_{pro}$). For species abbreviations and experimental details see **Extended Data Fig. 2A and 4**, respectively. Note the overlapping bacterial invasion zone and $PACE:NINmin_{pro}$*:GUS* expression in early infection stages (red and blue arrowheads in (**A - C**)). Red arrowheads: *M. loti Ds*Red. Blue arrowheads: GUS activity in nodule primordia. Only pictures taken under white light illumination (WLI) are displayed for nodules in panel VI to reveal the pink colour of leghemoglobin, characteristic for mature and fully infected nodules. Note that like *LjPACE*, the *PACE* variants-driven *GUS* expressions were absent at this stage (panel IV in (**A - C**) and panel IV in **Extended Data Fig. 4D**). (**D**) Quantification of transgenic root systems exhibiting *GUS* expression in different cell types and tissues exemplarily displayed in (**A - C**). n.d.: not determined. Data displayed in (**D**) are combined from two independent experiments. Bars, 250 µm.
